# α- and β-Casein Differentially Protect Anthocyanins from Three Red Grape (*Vitis vinifera* L.) Cultivars During Simulated Gastrointestinal Digestion

**DOI:** 10.3390/foods15183295

**Published:** 2026-09-17

**Authors:** Cosmin-Alin Faur, Adela Pintea, Dumitrița Rugină, Mihai Andrei Lăcătuș, Melinda Milena Horvat, Andrea Bunea

**Affiliations:** 1 Faculty of Animal Science and Biotechnology, University of Agricultural Sciences and Veterinary Medicine of Cluj-Napoca, Mănăștur 3–5, 400372 Cluj-Napoca, Romania; cosmin-alin.faur@usamvcluj.ro (C.-A.F.); mihai.lacatus@usamvcluj.ro (M.A.L.); 2 Faculty of Veterinary Medicine, University of Agricultural Sciences and Veterinary Medicine of Cluj-Napoca, Mănăștur 3–5, 400372 Cluj-Napoca, Romania; apintea@usamvcluj.ro (A.P.); dumitrita.rugina@usamvcluj.ro (D.R.); 3 Faculty of Medicine, University of Medicine and Pharmacy Iuliu Haţieganu Cluj-Napoca, Victor Babeş Street 8, 400012 Cluj-Napoca, Romania; melinda.horvat@umfcluj.ro

**Keywords:** grape, anthocyanins, casein, in vitro digestion, digestive recovery, cell viability

## Abstract

Grapes are valued for their high anthocyanin content, but the biological efficacy of these compounds is limited by their low digestive recovery. This study evaluated whether milk caseins improve the digestive recovery of anthocyanins from three red grape (*Vitis vinifera* L.) cultivars. Anthocyanins were profiled by HPLC-DAD-ESI-MS, total phenolic and flavonoid contents and antioxidant capacity were determined spectrophotometrically, the effect on cell viability was assessed on B16-F10 murine melanoma cells, and the digestive recovery of anthocyanins was measured after standardized static in vitro digestion (INFOGEST protocol) with and without α- or β-casein. Thirteen anthocyanins were detected, with malvidin-3-*O*-glucoside predominating in all cultivars. Fetească Neagră showed the highest total phenolic content (535.01 ± 38.43 mg gallic acid equivalents/100 g fresh weight), flavonoid content (202.52 ± 30.61 mg catechin equivalents/100 g) and antioxidant activity, and its extract reduced melanoma cell viability in a concentration-dependent manner, with viability remaining above 50% over the range tested. Total anthocyanin recovery during digestion decreased from 43.29–46.22% after the gastric phase to 8.82–12.25% after the intestinal phase. α-Casein significantly increased these values to 53.48–55.84% and 15.58–24.74%, respectively, in all cultivars. β-Casein had no significant effect in the gastric phase and was effective only in Pinot Noir after the intestinal phase (10.51% to 19.10%). α-Casein thus limited the intestinal degradation of grape anthocyanins, while cultivar composition determined the extent of the effect.

## 1. Introduction

Grapes are a carbohydrate-rich fruit providing minimal amounts of protein and fat, some essential micronutrients (vitamins C and K, carotenoid precursors of vitamin A, copper) and substantial levels of phenolic compounds, particularly anthocyanins [1]. These compounds have been linked to a reduced risk of several chronic diseases [2,3,4,5,6,7], and polyphenol-rich diets to a lower incidence of several cancers [8,9]. Extracts obtained from grape seeds, stems, skin, and pomace have shown antiproliferative and proapoptotic effects in colon, head and neck, renal, thyroid, breast, and melanoma models [10,11,12,13,14].

In *Vitis vinifera* L., anthocyanins occur mainly as 3-*O*-monoglucosides of delphinidin, cyanidin, petunidin, peonidin and malvidin, together with their acetyl-, *p*-coumaroyl- and caffeoyl-glucoside derivatives, malvidin-3-*O*-glucoside usually predominating [1]. These profiles are conventionally determined with acidified organic solvents, which maintain the stable flavylium form and maximise recovery from the skin tissue [15,16]; such values therefore describe the extractable rather than the physiologically released fraction. Anthocyanin behaviour is governed by a pH-dependent equilibrium between the flavylium cation, the quinoidal base, the colourless carbinol pseudobase and the chalcone, the latter forms being increasingly favoured as pH rises towards neutrality [17]. Anthocyanins are thus relatively stable in the stomach but undergo rapid hydration and ring opening in the small intestine, where degradation is further promoted by dissolved oxygen, digestive enzymes and bile salts [18,19]; acylated derivatives resist hydration better, owing to intramolecular copigmentation. This structural instability, rather than incomplete matrix release, is the principal reason why only a small fraction of ingested anthocyanins reaches the intestinal epithelium in an absorbable form [20], which limits their biological efficacy [21] and depends on their release and solubilization during digestion [22,23].

Bioaccessibility is defined as the fraction of an ingested compound released from the food matrix during digestion and made potentially available for intestinal absorption, whereas bioavailability further includes absorption, metabolism and systemic distribution [24]. Bioaccessibility therefore represents an upper limit for bioavailability and is the parameter estimated by in vitro digestion models, which are widely used because human studies are costly and ethically constrained [25]. In the present study, digestive recovery is defined as the percentage of a compound quantified in a given digestive fraction relative to its content in the undigested sample; it reflects stability during gastrointestinal digestion rather than availability for absorption [26]. Anthocyanins are among the flavonoids showing the lowest digestive recovery, frequently reported below 10% [27], with values for grapes ranging from 45% in the gastric digesta [28] to 7.6% after the intestinal phase [29]; recovery is influenced by temperature, pH, light exposure, and digestive conditions [26,30,31], as well as by interactions with other food components [32,33,34,35].

Milk proteins, particularly caseins, can stabilize anthocyanins by forming protein–anthocyanin complexes that limit degradation and promote gradual release along the gastrointestinal tract [36,37,38], with binding occurring through hydrophobic interactions and hydrogen bonding [39,40,41]. Caseins account for about 80% of bovine milk protein and comprise αs1-, αs2-, β- and κ-casein [42]; being intrinsically disordered proteins with open conformations and extensive solvent-exposed hydrophobic surfaces, they readily bind polyphenols [43]. α- and β-casein were selected here as the two most abundant fractions, which differ in the properties governing such binding—β-casein being the most hydrophobic and strongly amphipathic [44], αs1-casein less hydrophobic but more negatively charged—and because anthocyanin binding has been characterised for both [39,45,46]. κ-Casein, which is glycosylated, located at the micelle surface and rapidly cleaved by gastric proteases [47], may behave differently and was not investigated here. Protein binding is expected to contribute little in the oral and gastric phases, where the acidic pH keeps anthocyanins in the flavylium form, although caseins may already sequester part of the pigments and limit their contact with oxygen and enzymes [37,46]. The critical phase is the intestinal one, where caseins and the peptides released from them can shift the equilibrium away from the degradation-prone forms and act as a reservoir releasing the pigments gradually [39,45,48], thereby preserving a larger fraction potentially available for absorption [40].

Studies on fruit matrices have so far quantified mainly the total anthocyanin content, leaving the fraction recovered after digestion largely unexplored [37,49]. Anthocyanin–casein interactions have been investigated using blueberry extracts or unspecified grape skin material, so that the protective effect could not be related to a defined cultivar profile [39,40,46,49], while the available grape studies were conducted in different seasons and growing conditions, which complicates comparison between cultivars [28,29]. This is a relevant shortcoming, because both parameters are governed by several factors at once. Anthocyanin profiles are strongly cultivar-dependent, differing in the relative proportions of the individual 3-*O*-glucosides and in the extent of acylation [50], but are further modulated by the growing environment, with climate exerting the strongest influence, followed by soil type and water availability [51], so that the same cultivar can vary substantially between sites and seasons. Recovery during digestion is in turn determined by the conditions encountered along the gastrointestinal tract, principally the rise in pH but also temperature, oxygen exposure, and the presence of enzymes and bile salts [26,30,31], and by interactions with co-ingested food components [32,33,34,35]. Comparisons drawn across published studies therefore confound varietal, environmental and methodological contributions, which is why the three cultivars examined here were grown in the same vineyard, harvested in the same season and digested under a single standardised protocol. For Fetească Neagră, protein binding has been described with whey protein isolate but without assessment of digestive recovery [52], and reports on the antiproliferative activity of *V. vinifera* anthocyanins against the B16-F10 melanoma cell line remain scarce and have used grape pomace extracts [14,53,54].

We therefore hypothesised that α- and β-casein, differing in hydrophobicity and charge distribution, bind grape anthocyanins to different extents and consequently limit their degradation unequally during simulated digestion, the difference being most pronounced in the intestinal phase and modulated by the anthocyanin profile of the cultivar. The aim of this study was to investigate the impact of α- and β-casein on the stability and digestive recovery of anthocyanins from three red grape (*Vitis vinifera* L.) cultivars during simulated in vitro digestion: Fetească Neagră, an autochthonous Romanian cultivar for which no anthocyanin recovery during digestion data are available, together with Merlot and Pinot Noir as internationally distributed reference cultivars, all grown in the same vineyard and harvested in the same season. The extracts were first characterised in terms of physicochemical properties, total polyphenol, flavonoid and anthocyanin content, and antioxidant activity.

Digestion experiments quantify how much of a compound remains intact and soluble after passage through the gastrointestinal environment, but not whether that fraction retains biological activity. The extract of the cultivar richest in anthocyanins was therefore also tested on B16-F10 murine melanoma cells, a highly aggressive metastatic line widely used for the preliminary screening of plant extracts and one of the models in which grape-derived extracts have most consistently shown antiproliferative effects [10,11,12,13,14]. The two approaches are complementary: digestion defines the proportion of anthocyanins remaining in solution, while the cell assay establishes at what concentration an effect becomes detectable. The assay was performed on the undigested extract, since the digesta additionally contains caseins, enzymes, and bile salts that would preclude attributing any effect to the anthocyanins. To our knowledge, these four aspects (cultivar-specific anthocyanin profile, interaction with individual casein fractions, recovery during simulated digestion and effect on cell viability) have not previously been assessed on a single set of samples. Measuring all four on the same extracts, from vines of the same age grown in one vineyard and harvested in one season, allows the effect of each casein fraction to be attributed to a known anthocyanin composition rather than inferred across studies.

## 2. Materials and Methods

### 2.1. Plant Material

Grape berries from three red grape cultivars (*Vitis vinifera* L.), Fetească Neagră, Merlot, and Pinot Noir, were harvested in autumn 2024 from the experimental vineyards of the Faculty of Horticulture and Business in Rural Development in the Transylvania region. The grapevines were 11 years old, grafted, and maintained under uniform agronomic and phytosanitary conditions. Vines were trained on a single trellis system with a double trunk at 50 cm height, spur- and cane-pruned, and planted at a spacing of 1.10 m × 2.0 m. The vineyard followed no irrigation protocols. Standard vineyard management practices were employed throughout the growing season. For each cultivar, three independent samples of 200 berries were collected. Each sample comprised berries randomly taken from all ten vine plants and from different cluster positions and canopy exposures, so that within-vineyard and between-vine variability was integrated within every sample. The berries of each sample were pooled and homogenized separately, and the three resulting samples constituted the biological replicates (*n* = 3) used for all determinations and statistical comparisons. Harvesting was performed at optimal technological maturity for each cultivar, resulting in minor differences in harvest dates. The 2024 growing season was characterized by high temperatures and uneven precipitation. Sampling was conducted between late September and early October 2024. After harvest, samples were transported to the Department of Chemistry and Biochemistry and stored at −80 °C until analysis.

Previous studies have shown that prolonged frozen storage may affect certain quality parameters of plant-derived samples [55]. To limit potential storage-related variability, all three biological replicates of each cultivar were processed in parallel under identical analytical conditions, with samples kept at −80 °C from harvest until analysis. Storage duration was therefore equivalent across replicates and cultivars, ensuring that comparisons among cultivars were not confounded by differences in storage time.

### 2.2. Chemical Reagents

All solvents used for extraction and chromatographic analyses, including methanol, chloroform, ethyl acetate, ethanol, formic acid, trifluoroacetic acid (TFA), and acetonitrile, were purchased from Merck (Darmstadt, Germany). Folin–Ciocâlteu reagent was obtained from VWR International (Radnor, PA, USA). Standards for phenolic compounds, including malvidin-3-*O*-glucoside (Polyphenols, Sandnes, Norway), gallic acid, and catechin (Sigma-Aldrich, St. Louis, MO, USA), were used for calibration and quantification. Amberlite XAD-7 and Sephadex LH-20 (Sigma-Aldrich) were employed for purification procedures. Enzymes and digestion reagents, including α-amylase (A1031), pepsin from porcine gastric mucosa (P6887), pancreatin from porcine pancreas (P7545), bile salts (B8756), as well as α-casein (C6780; from bovine milk, lyophilized powder, ≥70% αs-casein basis by electrophoresis, consisting of αs1- and αs2-casein) and β-casein (C6905; from bovine milk, BioUltra grade, ≥98% by PAGE, essentially salt-free lyophilized powder), were obtained from Sigma-Aldrich. For cell culture assays, Dulbecco’s Modified Eagle Medium (DMEM), fetal bovine serum (FBS), glutamine, and antibiotics were purchased from Gibco (Carlsbad, CA, USA), while the WST-1 cell proliferation reagent was obtained from Sigma-Aldrich. All solutions were prepared using bidistilled water.

### 2.3. Physicochemical Parameters of Grapes

The physicochemical characteristics of grape samples, including pH and titratable acidity, were determined according to the official methods of the International Organisation of Vine and Wine [56].

### 2.4. Preparation of Crude Methanolic Extract

The methanolic extraction from grape berries was carried out following a modified version of previously published procedures [15,16]. The berry skins (1 g) from three red grape cultivars were manually separated from the pulp and seeds, and subsequently homogenized in acidified methanol (0.3% HCl, *v*/*v*) using a Miccra D-9 KT Digitronic homogenizer (Bergheim, Germany). Extraction was repeated several times until the solvent became colourless, with the final extraction step performed overnight at 4 °C in the dark. Acidified methanol was selected because anthocyanins are polar glycosides whose extraction from skin tissue is most efficient in polar organic solvents, methanol giving higher recoveries than ethanol or acetone under comparable conditions [16]. Acidification serves a distinct purpose: at low pH the flavylium cation predominates, which is both the most stable form and the one most soluble in the extraction medium, so that conversion to the carbinol pseudobase and chalcone during extraction is suppressed [17]. As this solvent is not food-grade, the extracts are analytical rather than food preparations, and the anthocyanin profile obtained represents the maximum extractable fraction rather than the fraction released under physiological conditions. The resulting extracts were filtered through multiple layers of cotton wool and evaporated under reduced pressure at 35 °C to completely remove methanol.

### 2.5. Determination of Total Phenolic Content

Total phenolic content (TPC) was determined using the Folin–Ciocalteu assay according to the method originally described by Singleton et al. [57], with the procedure adapted for grape matrices based on the methodology reported by Abreu et al. [58]. Methanolic extracts (25 μL) were reacted with Folin–Ciocalteu reagent (120 μL) and Na_2_CO_3_ (340 μL), and absorbance was measured at 750 nm after 90 min incubation in the dark. Results were expressed as mg gallic acid equivalents (GAE)/100 g fresh weight (FW) using a gallic acid calibration curve (R^2^ = 0.997).

### 2.6. Determination of Total Flavonoid Content

Total flavonoid content (TFC) was determined using the aluminium chloride colorimetric assay in the sequential NaNO_2_–AlCl_3_–NaOH variant, based on the previously described protocols for citrus fruits [59] and strawberries [60]. Minor modifications were introduced to adapt the procedure to grape extracts. The extract was diluted to 5 mL, reacted sequentially with NaNO_2_, AlCl_3_, and NaOH, and absorbance was measured at 510 nm. Results were expressed as mg catechin equivalents (CE)/100 g FW using a catechin calibration curve.

### 2.7. Antioxidant Activity Assays

#### 2.7.1. 2,2-Diphenyl-1-Picrylhydrazyl Radical Scavenging (DPPH) Assay

Antioxidant activity was evaluated using the DPPH assay based on the procedure described by Brand-Williams et al. [61] and applied to grape pomace extracts by Karastergiou et al. [62], with minor adaptations for the grape samples analyzed in the present study. Samples were mixed with DPPH solution, incubated at 40 °C for 30 min, and absorbance was measured at 517 nm. Results were expressed as mmol Trolox equivalents (TE)/g FW using a Trolox calibration curve (R^2^ = 0.9972).

#### 2.7.2. 2,2′-Azino-Bis(3-Ethylbenzothiazoline-6-Sulfonic Acid) Radical Cation Scavenging (ABTS/TEAC) Assay

Antioxidant capacity was determined using the ABTS assay based on the procedure described by Re et al. [63], with ABTS•^+^ solution standardized as reported for grape extracts by Karastergiou et al. [62]. The ABTS•^+^ solution was prepared, diluted to an absorbance of 0.70 ± 0.05 at 734 nm, and reacted with the sample, followed by incubation for 6 min at 30 °C. Absorbance was measured at 734 nm, and results were expressed as mmol TE/g FW using a Trolox calibration curve (R^2^ = 0.9969).

#### 2.7.3. Oxygen Radical Absorbance Capacity (ORAC) Assay

Antioxidant activity was evaluated using the ORAC assay based on the procedure described by Huang et al. [64], as applied to grape matrices by Karastergiou et al. [62]. Fluorescein and the sample were incubated at 37 °C, followed by AAPH addition, and fluorescence decay was monitored (Ex 480 nm, Em 525 nm). Results were expressed as mmol TE/g FW using a Trolox calibration curve (R^2^ = 0.9909).

#### 2.7.4. Ferric Reducing Antioxidant Power (FRAP) Assay

Reducing power was determined using the FRAP assay based on the procedure described by Benzie and Strain [65], as applied to grape matrices by Karastergiou et al. [62]. FRAP reagent was mixed with the sample, incubated for 3 min at room temperature, and absorbance was measured at 593 nm. Results were expressed as mmol Fe^2+^ equivalents/g FW using an FeSO_4_ calibration curve (R^2^ = 0.9997).

### 2.8. Isolation, Purification and Determination of Anthocyanins

#### 2.8.1. Preparation of Anthocyanin Fraction

The anthocyanin-rich fraction was prepared according to the procedure previously reported by Faur et al. [50]. Briefly, the obtained methanolic extract was purified by liquid–liquid extraction with ethyl acetate and water. The remaining aqueous phase was subsequently loaded through an Amberlite XAD-7 column (1 × 0.5 cm) conditioned with six column volumes of 0.3% aqueous trifluoroacetic acid (TFA). After sample loading, the column was washed with four volumes of 0.3% aqueous TFA to eliminate sugars, pectins, and other free impurities. The elution of anthocyanins and procyanidins was achieved using four column volumes of methanol containing 0.3% TFA (*v*/*v*). The extract was further purified on a Sephadex LH-20 column (2.5 × 0.5 cm), previously conditioned and eluted with ten volumes of a water–methanol mixture (8:2, *v*/*v*) containing 0.3% TFA. The resulting anthocyanin-rich fraction was adjusted to a final volume of 5 mL with distilled water, filtered through a 0.45 µm membrane, and analyzed by high-performance liquid chromatography coupled with diode array detection (HPLC-DAD, Prominence series, Shimadzu Corporation, Kyoto, Japan) and electrospray ionization mass spectrometry (HPLC-ESI-MS, Agilent Technologies, Santa Clara, CA, USA).

#### 2.8.2. HPLC-DAD Analysis of Anthocyanins

HPLC-DAD analysis was performed according to our previously reported procedure [50]. Separation and quantification of anthocyanins were conducted using a Shimadzu high-performance liquid chromatography system (Prominence series, Shimadzu Corporation, Kyoto, Japan) equipped with an LC-20AT binary pump, a DGU-20A3 degasser, a set of SPD-M20 diode array detectors. Separation was achieved on a Luna Phenomenex C18 column (5 µm, 250 × 4.6 mm). The mobile phase consisted of solvent A (4.5% formic acid in distilled water) and solvent B (acetonitrile). The gradient program started with 10% solvent B maintained for 9 min, followed by a linear increase to 12% at 17 min and to 25% at 30 min. From 30 to 50 min, solvent B increased to 90%. The flow rate was maintained at 0.8 mL/min, and all analyses were conducted at 35 °C. Quantification of anthocyanins was performed by external calibration, using malvidin-3-*O*-glucoside as standard, within the concentration range of 2.5–500 µg/mL. Chromatograms were recorded at 520 nm. The total anthocyanin content was expressed as the sum of all identified anthocyanins and reported as mg malvidin-3-*O*-glucoside equivalent (MGE)/100 g FW.

#### 2.8.3. Identification of Individual Anthocyanins by LC-MS-ESI+

Mass spectrometry (MS) analysis was performed using an Agilent 1200 HPLC system equipped with a quaternary pump, solvent degasser, autosampler, UV-VIS/DAD and coupled with a single-quadrupole mass detector Agilent model 6110 (Agilent Technologies, Santa Clara, CA, USA), according to our previously reported procedure [50]. Separation was achieved on an Eclipse XDB C18 column (5 µm, 4.6 × 150 mm, Agilent Technologies, Santa Clara, CA, USA). The mobile phases consisted of solvent C (water containing 0.1% acetic acid) and solvent D (acetonitrile containing 0.1% acetic acid). Elution was performed using a gradient program at a flow rate of 0.5 mL/min and a column temperature of 25 °C for a total run time of 30 min. The gradient (in % solvent D) was as follows: 0–2 min, 5%; 2–18 min, 5–40%; 18–20 min, 40–90%; 20–24 min, 90%; 24–25 min, 90–5%; and 25–30 min, 5%. Chromatograms were recorded at 280, 340, and 520 nm. For MS, positive electrospray ionization mode was utilized under the following conditions: capillary voltage 3000 V, temperature 350 °C, nitrogen flow rate 7 L/min, nebulization pressure 35 psi, fragmentor voltage 100 eV and *m*/*z* range 120–1200 in full-scan mode. Data acquisition and result interpretation were carried out using Agilent ChemStation software (Version B.04.02 SP1).

### 2.9. Cell Culture and Cell Viability Assay

The anthocyanin extract obtained from the cultivar with the highest anthocyanin content (Fetească Neagră) was selected for further analysis of its effect on the viability of a tumour cell line at different concentrations. The undigested extract was used for viability assay, so that the observed effect could be attributed to the anthocyanins themselves rather than to the additional components present in the digesta. The experiments were performed on a B16-F10 metastatic murine melanoma (skin melanoma, ATCC^®^ CRL-6475) cell line stored in liquid nitrogen (−196 °C) at the Institute of Life Sciences Cluj-Napoca and cultivated under standard conditions according to the procedure previously described by our group [66] at 37 °C, 5% (*v*/*v*) CO_2_, and 95% (*v*/*v*) relative humidity, in Dulbecco’s Modified Eagle’s Medium containing 2 mM L-glutamine, antibiotics (a mix of penicillin and streptomycin, 1%), and 10% (*v*/*v*) fetal bovine serum. Cell viability was assessed using the WST-1 colorimetric assay [67].

The B16-F10 cells were seeded in a 96-well microplate at a rate of 8 × 10^3^ per well and incubated overnight. The next day, the B16-F10 cells were treated with different concentrations of the grape extract (0–10 µg malvidin-3-*O*-glucoside/mL extract). After a 24 h incubation period, the WST-1 reagent (150 μL/well) was applied to the treated B16-F10 cells for 30 min. Following the observed colorimetric reaction, the absorbance of the samples was measured at 450 nm with an HT BioTek Synergy microplate reader (BioTek Instruments, Winooski, VT, USA). Cell viability was expressed as a percentage of the control group, which consisted exclusively of cells cultured in normal conditions.

### 2.10. Simulated In Vitro Digestion

Simulated gastrointestinal digestion was performed according to the standardized INFOGEST protocol [25], with slight modifications, as described below. Digestion was applied to the crude methanolic extract and not to intact grape skin, so that a constant and identically prepared anthocyanin load was delivered to all digestion vessels of a given cultivar, and the effect of casein addition could be isolated from matrix-release phenomena. Each phase was prepared with the corresponding simulated fluids, specific enzymes, CaCl_2_ for enzyme activation, and bidistilled water to maintain the required concentrations. Simulated fluids were prepared as 1.25-fold concentrated electrolyte stock solutions according to the INFOGEST protocol. CaCl_2_ was added at the beginning of each phase from a 0.03 M stock solution, giving final concentrations of 0.75 mM in the oral, 0.075 mM in the gastric, and 0.3 mM in the intestinal phase. All enzyme activities are expressed as final activities in the corresponding digestion mixture and not as activities of the added stock solutions: 75 U/mL for salivary α-amylase in the oral phase, 2000 U/mL for pepsin in the gastric phase and 100 U/mL, based on trypsin activity, for pancreatin in the intestinal phase. Bile salts were added to a final concentration of 0.01 M (10 mM) in the intestinal phase. The pH was adjusted with HCl (6 M) or NaOH (1 M). Digestions were carried out at 37 °C (±0.5 °C) under static conditions with continuous linear shaking at the highest setting of the shaking device, corresponding to approximately 160 strokes per minute with a stroke length of approximately 15 mm (WNE 14 water bath fitted with an SV 1422 shaking device, Memmert GmbH + Co. KG, Schwabach, Germany).

Although a 2 min simulated oral phase was performed according to the standardized INFOGEST protocol, anthocyanins were not analyzed at this stage. As noted by Minekus et al., digestion time in the oral cavity is very short, and most liquids do not require an oral phase, particularly for compounds not affected by salivary enzymes [25]. The oral phase was initiated by adding 4 mL of grape extract at a concentration of 0.2 mg/mL, obtained by methanol extraction, followed by solvent evaporation and resuspension in bidistilled water, to 2.7 mL simulated salivary fluid (SSF), 0.5 mL α-amylase, 200 µL CaCl_2_, and 600 µL bidistilled water to a final volume of 8 mL, followed by incubation for 2 min at pH 7.0. The gastric phase was initiated by adding the entire oral digesta to 5.1 mL simulated gastric fluid (SGF), 1.3 mL pepsin solution, and 40 µL CaCl_2_; the pH was adjusted to 3.0, and the final volume was brought to 16 mL with water, followed by incubation for 2 h. The intestinal phase was then initiated by adding the gastric digesta to 6.2 mL simulated intestinal fluid (SIF), 3.3 mL pancreatin solution, 3.3 mL bile salts and 320 µL CaCl_2_; the pH was adjusted to 7.0 and the final volume to 32 mL with water, then incubated for 2 h. To evaluate the effect of caseins, α-casein or β-casein was added to the anthocyanin extract prior to digestion. The casein solutions were prepared in simulated salivary fluid at a concentration of 0.8 mg/mL, and 1 mL was introduced into the oral phase in place of an identical volume of SSF, giving a final casein concentration of 0.1 mg/mL in the oral phase. Since the casein was dissolved in the same electrolyte solution that it replaced, both the total volume of the oral phase (8 mL) and its electrolyte composition remained identical to those of the casein-free controls. As 4 mL of extract at 0.2 mg/mL were introduced into the same final volume, the extract also reached 0.1 mg/mL, corresponding to a casein-to-extract mass ratio of approximately 1:1 (0.8 mg of each). This concentration was selected based on previous studies investigating casein–anthocyanin interactions during simulated gastrointestinal digestion, which used the same protein concentration in comparable digestion systems [39,45], which allows a direct comparison of our results with published data; it should be noted that this value is well below the casein content of bovine milk (≈25–28 mg/mL) [68] and was not intended to reproduce a dairy matrix, but to provide a defined and reproducible protein background. The concentration dependence of the observed effect was not investigated in the present study. After each digestion phase, the enzymatic reaction was stopped by cooling on ice and the digesta was acidified to pH 3.0 with 6 M HCl, in order to convert the anthocyanins to the stable flavylium form and to bring all samples to a common, defined condition prior to quantification. The same acidification was applied to every sample, including the casein-free controls. During this adjustment, the pH passes through the isoelectric region of casein (pI ≈ 4.6), and the pellet fraction was not analysed; anthocyanins lost through chemical degradation can therefore not be distinguished from those lost through insolubilization or co-precipitation with protein. The values reported consequently describe the anthocyanin fraction recovered after digestion, and do not constitute a mass balance. Samples were then centrifuged (9000× *g*, 50 min, 4 °C), and the entire supernatant was collected, evaporated to dryness under reduced pressure and redissolved in 2 mL of bidistilled water, before filtration through a 0.22 µm membrane and analysis by HPLC.

Digestive recovery was calculated as:

Digestive recovery (%) = (C_digesta/C_control) × 100

where C_digesta is the anthocyanin concentration determined by HPLC in the acid-soluble fraction recovered after the gastric or intestinal phase, and C_control is the concentration measured in an undigested control prepared in parallel. The same calculation was applied to total and to individual anthocyanins, and identical processing was used for the casein-containing samples and their casein-free controls.

### 2.11. Statistical Analysis

Values are presented as mean ± SD of three independent experiments. For cell cultures, each replicate corresponds to an average of five wells per treatment or control. Initial data exploration, including calculation of descriptive statistics such as mean, median, and standard deviation, was performed using Microsoft Excel 365. Subsequent statistical analyses were conducted using IBM SPSS Statistics (Version 26), XLSTAT (Version 2019.2.2), and GraphPad Prism (Version 8). Prior to analysis of variance, the assumptions of normality and homogeneity of variances were verified using the Shapiro–Wilk and Levene tests, respectively. Data was screened for outliers by inspection of studentised residuals. No observation exceeded ±3 standard deviations and no data points were excluded. Differences among groups were evaluated using one-way and two-way ANOVA. For the digestion experiments, Duncan’s Multiple Range Test was applied as an all-pairwise comparison among the six treatment × phase groups, so that the control, α-casein and β-casein treatments are compared directly with one another. For the cell viability assay, in which all concentrations are compared with a single untreated control, Dunnett’s multiple comparisons test was used. Significance was set at *p* < 0.05. Multivariate analysis was performed using Principal Component Analysis (PCA) to reduce dimensionality and highlight correlations among the measured variables, namely total polyphenol, flavonoid and anthocyanin content, the four antioxidant capacity assays (DPPH, ABTS, ORAC, FRAP) and the five major individual anthocyanins. The purpose was twofold: to establish which antioxidant assays are driven by the anthocyanin fraction and which respond to other phenolic constituents, and to obtain an integrated compositional description of each cultivar that could subsequently be related to its behaviour during digestion. Variables were mean-centered and scaled to unit variance prior to analysis to ensure comparability among measurements expressed in different units.

## 3. Results

### 3.1. Physicochemical Parameters

No statistically significant differences were found among the three cultivars for either pH or titratable acidity (*p* > 0.05, Table 1). Values ranged from 3.30 ± 0.45 to 3.60 ± 0.37 for pH, the highest being recorded in Fetească Neagră, and from 4.30 ± 0.66 to 6.20 ± 0.24 g L^−1^ for titratable acidity, the highest being recorded in Pinot Noir.

### 3.2. Determination of Total Anthocyanins, Polyphenols and Flavonoids

Total anthocyanin, polyphenol and flavonoid contents differed significantly among the three cultivars (*p* < 0.05, Table 2). The highest values for all three parameters were recorded in Fetească Neagră, while Pinot Noir exhibited the lowest concentrations.

### 3.3. Antioxidant Activity

The antioxidant activity of the three cultivars was evaluated by four assays (Table 3). In the DPPH assay, Fetească Neagră showed significantly higher activity than Pinot Noir, while Merlot did not differ significantly from either cultivar. The same ranking was obtained by ABTS and FRAP, with Fetească Neagră recording significantly higher values than both other cultivars, whereas no significant differences were found in the ORAC assay.

### 3.4. Identification and Quantification of Anthocyanins in Grapes

**Figure 1:**
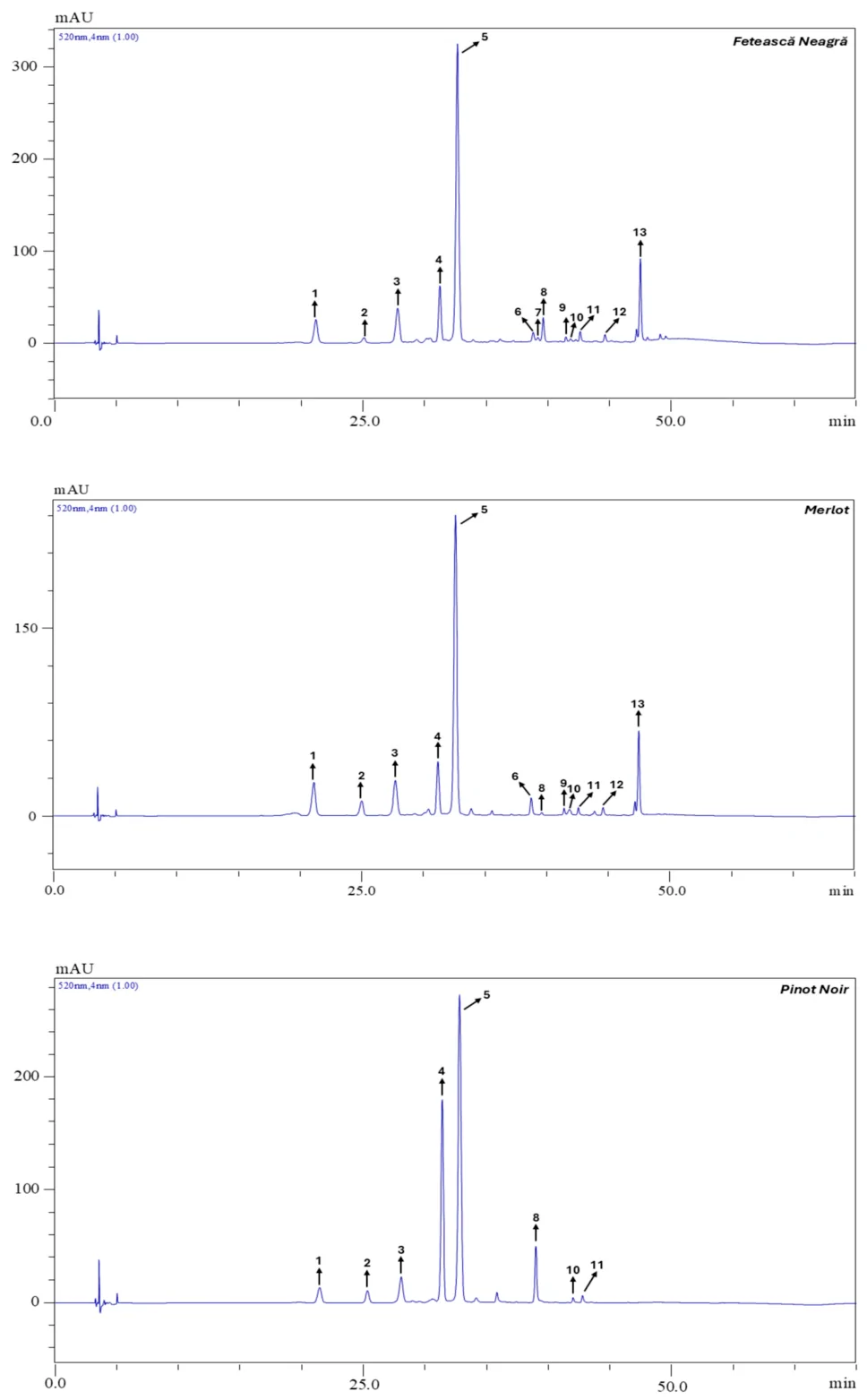
The HPLC chromatogram for anthocyanin separation in analyzed red grape cultivars (*V. vinifera* L.). (1) delphinidin-3-*O*-glucoside; (2) cyanidin-3-*O*-glucoside; (3) petunidin-3-*O*-glucoside; (4) peonidin-3-*O*-glucoside; (5) malvidin-3-*O*-glucoside; (6) petunidin-3-*O*-acetylglucoside; (7) delphinidin-3-*O*-*p*-coumaroylglucoside; (8) peonidin-3-*O*-acetylglucoside; (9) malvidin-3-*O*-acetylglucoside; (10) cyanidin-3-*O-p*-coumaroylglucoside; (11) petunidin-3-*O*-*p*-coumaroylglucoside; (12) peonidin-3-*O*-*p*-coumaroylglucoside; (13) malvidin-3-*O*-*p*-coumaroylglucoside (peak numbers correspond with those from Table 4). The identification of individual anthocyanins was made based on HPLC-ESI-MS.

Thirteen individual anthocyanins were identified based on retention times and mass spectrometric data (Table 4), with all thirteen being detected in Fetească Neagră, twelve in Merlot and eight in Pinot Noir. Representative HPLC chromatograms recorded at 520 nm are shown in Figure 1, while the individual chromatograms of each cultivar, at higher magnification, are provided as Supplementary Materials (Figures S1–S3). The three cultivars differed markedly in the distribution of anthocyanin classes: monoglucosides accounted for 80.5%, 82.7% and 92.4% of total anthocyanins in Fetească Neagră, Merlot and Pinot Noir, and acylated forms for 19.5%, 17.3% and 7.6%, respectively. This difference was driven almost entirely by the *p*-coumaroylated fraction, which reached 16.7% in Fetească Neagră and 13.3% in Merlot but only 2.7% in Pinot Noir, where malvidin-3-*O*-*p*-coumaroylglucoside was not detected.

Malvidin-3-*O*-glucoside was the predominant compound in all cultivars, followed by peonidin-3-*O*-glucoside, malvidin-3-*O*-*p*-coumaroylglucoside and petunidin-3-*O*-glucoside (Table 5). Pinot Noir was distinguished by a markedly higher proportion of peonidin-3-*O*-glucoside, which was present at a significantly higher concentration in this cultivar than in the other two, whereas delphinidin-3-*O*-*p*-coumaroylglucoside, the least abundant compound overall, was detected only in Fetească Neagră. Most individual anthocyanins occurred at significantly higher concentrations in Fetească Neagră.

### 3.5. Effect of the Anthocyanin Extract on the Viability of B16-F10 Melanoma Cells

**Figure 2:**
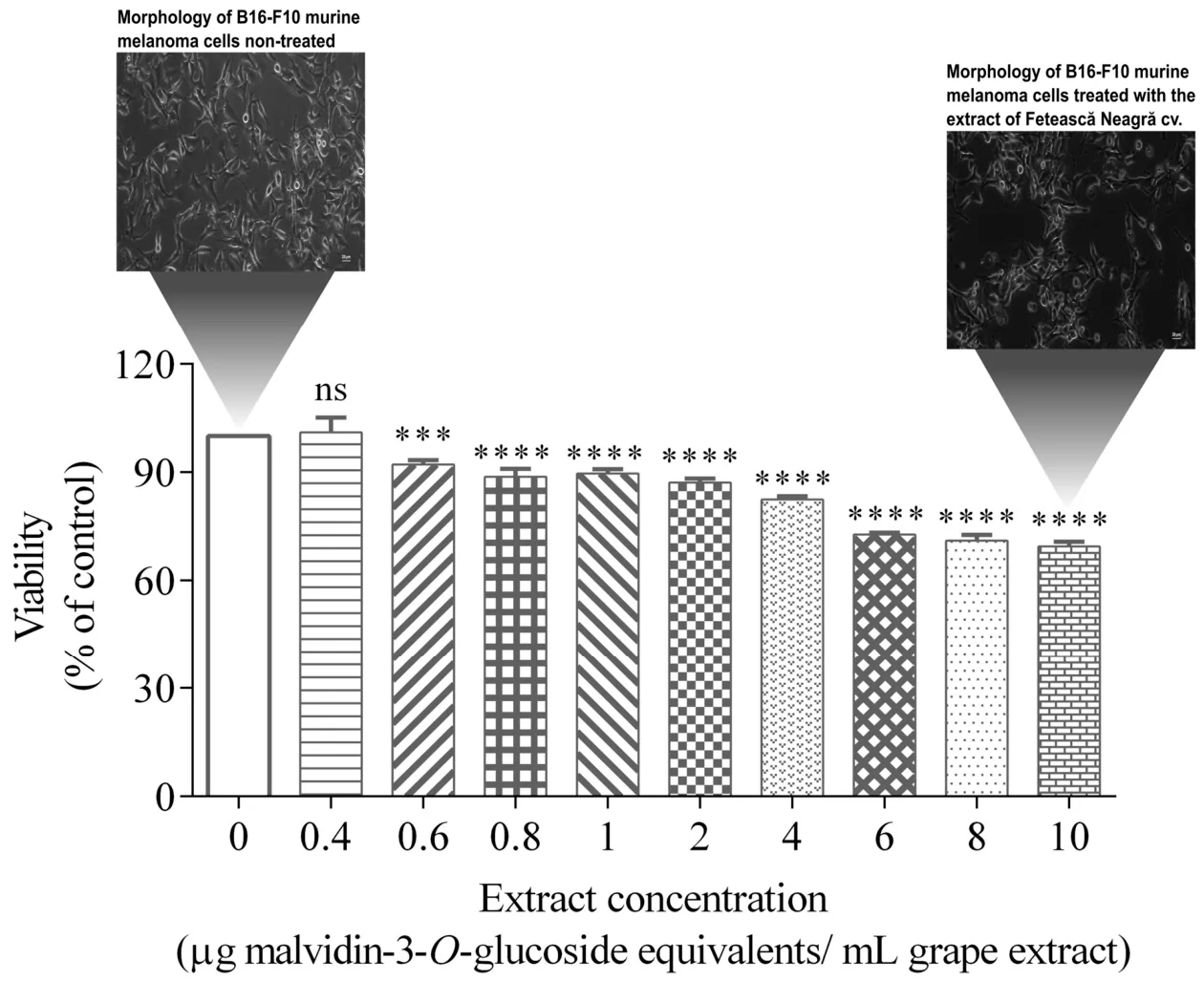
Viability of B16-F10 murine melanoma cells treated for 24 h with different concentrations of the anthocyanin-rich extract of Fetească Neagră (0–10 µg malvidin-3-*O*-glucoside equivalents/mL of extract). Data are expressed as a percentage of the untreated control (100% viability) and represent means of three independent experiments, each performed with five replicate wells per concentration. Scale bar = 20 µm. Statistical significance versus control: ns = not significant, *** *p* < 0.001, **** *p* < 0.0001 (one-way ANOVA, Dunnett’s multiple comparisons test).

B16-F10 cells were treated for 24 h with the anthocyanin-rich extract of Fetească Neagră at concentrations of 0–10 µg malvidin-3-*O*-glucoside equivalents/mL extract. Cell viability decreased in a concentration-dependent manner with respect to untreated control cells (Figure 2). Higher-magnification micrographs of cell morphology are provided in the Supplementary Materials (Figures S4 and S5). No effect was observed at 0.4 µg/mL, whereas concentrations from 0.6 µg/mL upwards reduced viability by up to 30%.

### 3.6. Anthocyanin Recovery During Simulated Gastrointestinal Digestion

Anthocyanin recovery during digestion was expressed as the percentage of anthocyanins recovered in the soluble fraction at the end of the gastric and intestinal phases, relative to the amount introduced at the beginning of the oral phase (Table S1). Soluble anthocyanin recovery depended on both the cultivar and the digestion phase: values ranged from 43.29 to 46.22% after the gastric phase and from 8.82 to 12.25% after the intestinal phase, with the highest gastric value being recorded for Merlot (46.22 ± 4.50%) and the highest intestinal value for Fetească Neagră (12.25 ± 2.18%).

Digestive recovery values for individual anthocyanins were generally comparable among cultivars. After the gastric phase, the highest values were consistently recorded for peonidin-3-*O*-glucoside, exceeding 58% in all three cultivars, and the lowest for the *p*-coumaroylated derivatives. The pattern shifted after the intestinal phase, where the highest values were obtained for acylated compounds (delphinidin-3-*O*-*p*-coumaroylglucoside in Fetească Neagră, petunidin-3-*O*-acetylglucoside in Merlot and cyanidin-3-*O*-*p*-coumaroylglucoside in Pinot Noir), whereas delphinidin-3-*O*-glucoside showed the lowest values in all three cultivars.

### 3.7. Effect of α- and β-Casein on Anthocyanin Recovery

**Figure 3:**
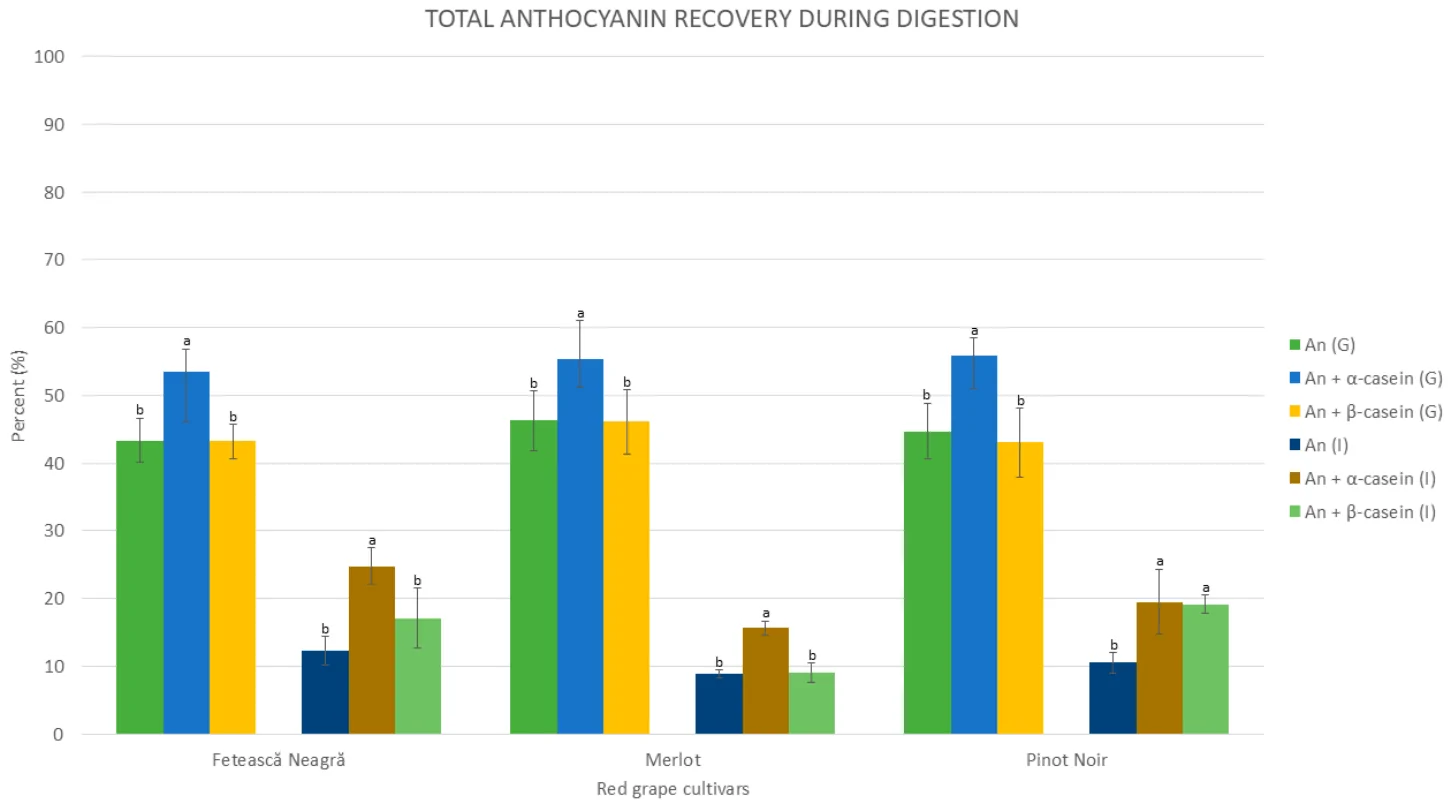
Total anthocyanin recovery during simulated in vitro digestion (%) of red grape extracts after the gastric and intestinal phases, in the absence of casein (control) and in the presence of α-casein or β-casein (0.1 mg/mL). Values are expressed as the percentage of the anthocyanins recovered in the soluble fraction relative to the undigested extract, taken as 100%. Data are means ± standard deviation of three independent digestions. Different letters above the bars indicate significant differences at *p* < 0.05 (two-way ANOVA followed by Duncan’s Multiple Range Test).

α-Casein significantly increased the recovery of total anthocyanins in all three cultivars, by 9.1–11.2 percentage points after the gastric phase and by 6.8–12.5 percentage points after the intestinal phase, the latter corresponding to a 77–102% increase over the corresponding controls (Figure 3). β-Casein had no significant effect in the gastric phase. After the intestinal phase, it produced a significant increase only in Pinot Noir, from 10.51 ± 1.56% to 19.10 ± 1.33%, with no significant differences from α-casein.

**Figure 4:**
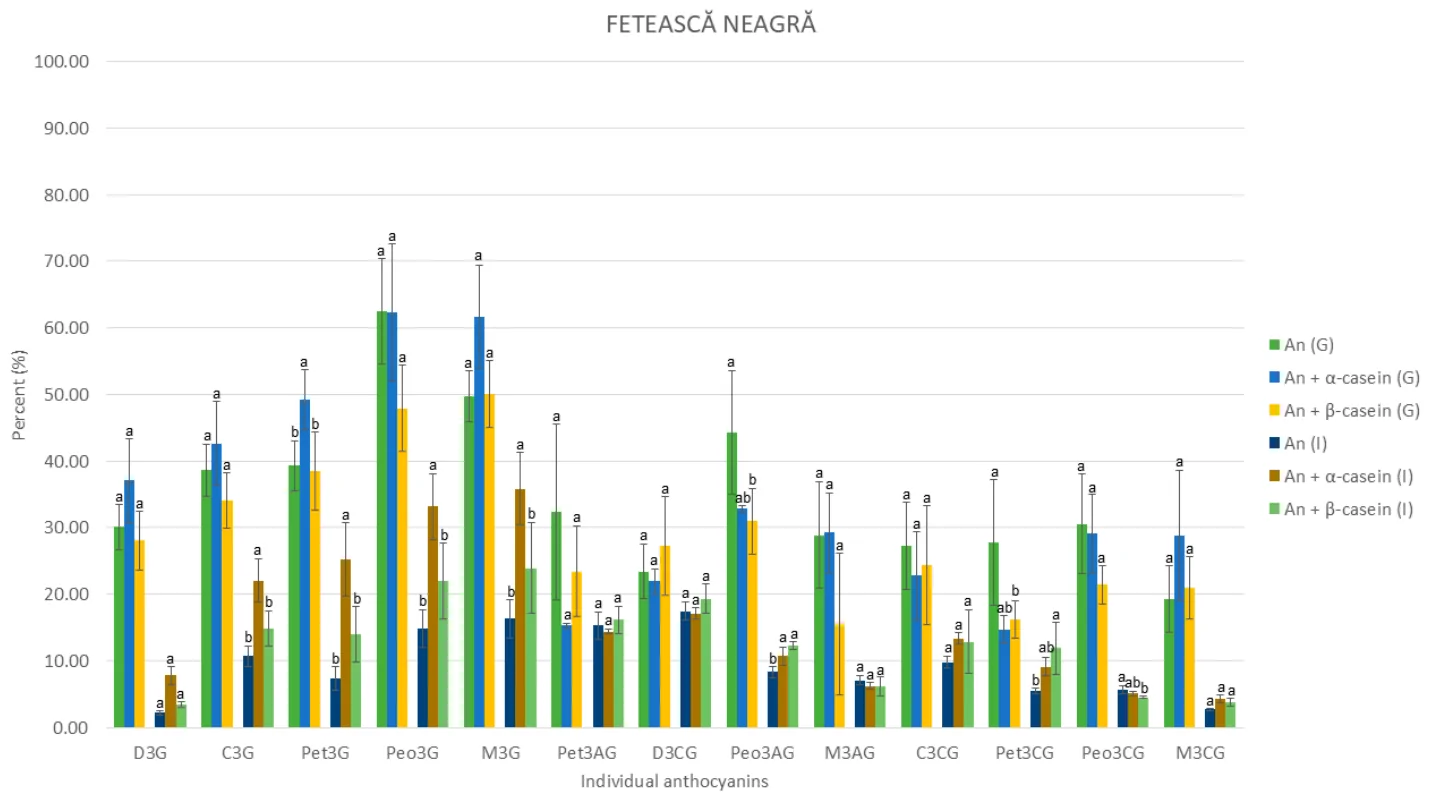
Digestive recovery (%) of individual anthocyanins in the Fetească Neagră grape extract after the gastric and intestinal phases of simulated in vitro digestion, in the absence of casein (control) and in the presence of α-casein or β-casein (0.1 mg/mL). Values are expressed as the percentage of each anthocyanin recovered in the soluble fraction relative to the undigested extract, taken as 100%. Data are means ± standard deviation of three independent digestions. Different letters above the bars indicate significant differences at *p* < 0.05 (two-way ANOVA followed by Duncan’s Multiple Range Test). D3G, delphinidin-3-*O*-glucoside; C3G, cyanidin-3-*O*-glucoside; Pet3G, petunidin-3-*O*-glucoside; Peo3G, peonidin-3-*O*-glucoside; M3G, malvidin-3-*O*-glucoside; Pet3AG, petunidin-3-*O*-acetylglucoside; D3CG, delphinidin-3-*O*-*p*-coumaroylglucoside; Peo3AG, peonidin-3-*O*-acetylglucoside; M3AG, malvidin-3-*O*-acetylglucoside; C3CG, cyanidin-3-*O*-*p*-coumaroylglucoside; Pet3CG, petunidin-3-*O*-*p*-coumaroylglucoside; Peo3CG, peonidin-3-*O*-*p*-coumaroylglucoside; M3CG, malvidin-3-*O*-*p*-coumaroylglucoside.

**Figure 5:**
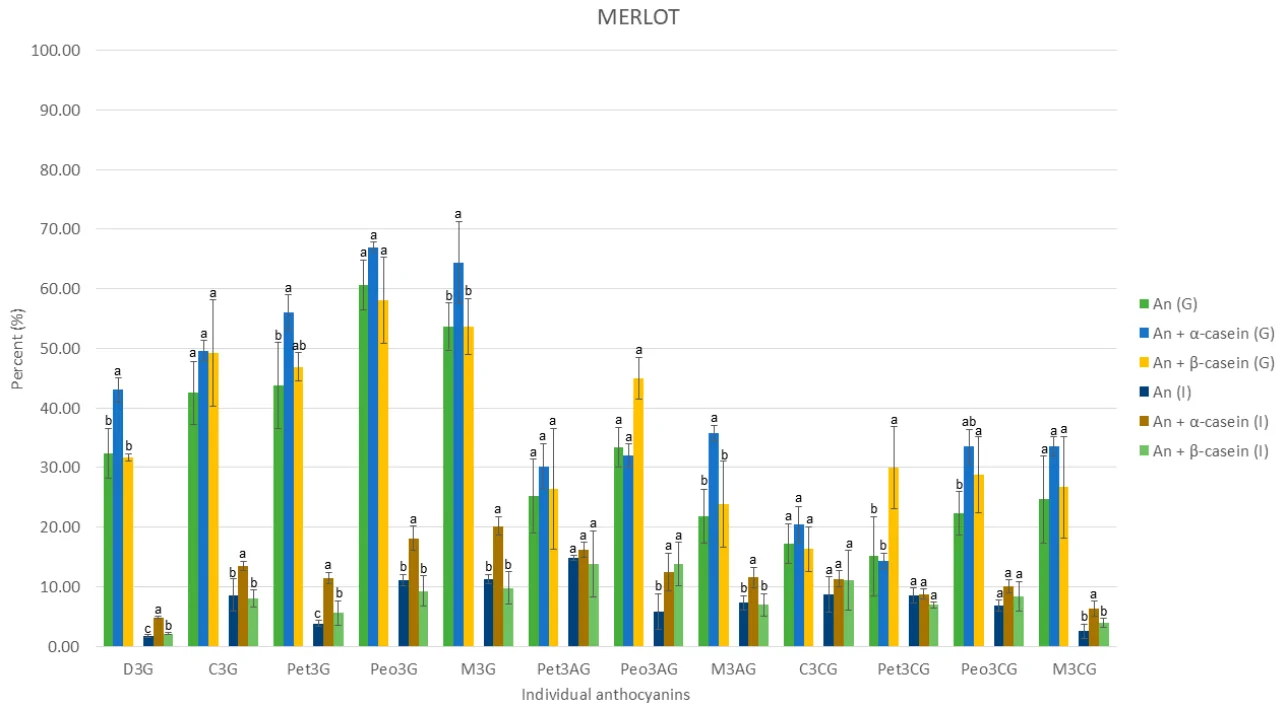
Digestive recovery (%) of individual anthocyanins in the Merlot grape extract after the gastric and intestinal phases of simulated in vitro digestion, in the absence of casein (control) and in the presence of α-casein or β-casein (0.1 mg/mL). Values are expressed as the percentage of each anthocyanin recovered in the soluble fraction relative to the undigested extract, taken as 100%. Data are means ± standard deviation of three independent digestions. Different letters above the bars indicate significant differences at *p* < 0.05 (two-way ANOVA followed by Duncan’s Multiple Range Test). D3G, delphinidin-3-*O*-glucoside; C3G, cyanidin-3-*O*-glucoside; Pet3G, petunidin-3-*O*-glucoside; Peo3G, peonidin-3-*O*-glucoside; M3G, malvidin-3-*O*-glucoside; Pet3AG, petunidin-3-*O*-acetylglucoside; Peo3AG, peonidin-3-*O*-acetylglucoside; M3AG, malvidin-3-*O*-acetylglucoside; C3CG, cyanidin-3-*O*-*p*-coumaroylglucoside; Pet3CG, petunidin-3-*O*-*p*-coumaroylglucoside; Peo3CG, peonidin-3-*O*-*p*-coumaroylglucoside; M3CG, malvidin-3-*O*-*p*-coumaroylglucoside.

**Figure 6:**
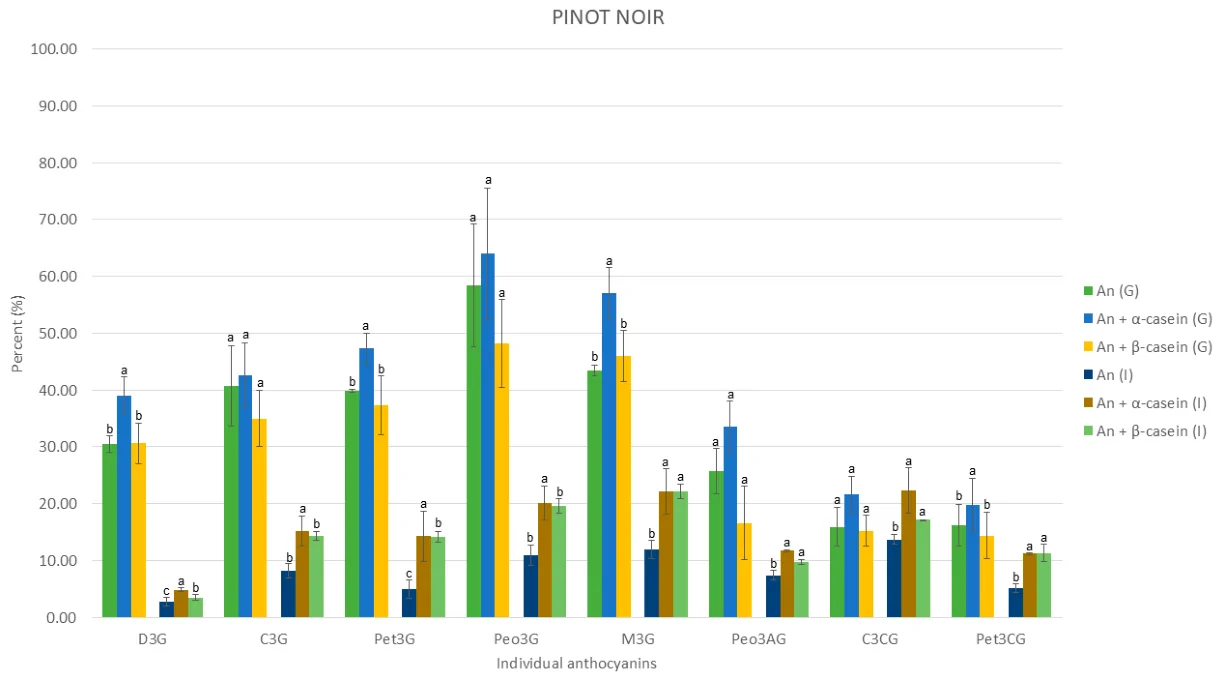
Digestive recovery (%) of individual anthocyanins in the Pinot Noir grape extract after the gastric and intestinal phases of simulated in vitro digestion, in the absence of casein (control) and in the presence of α-casein or β-casein (0.1 mg/mL). Values are expressed as the percentage of each anthocyanin recovered in the soluble fraction relative to the undigested extract, taken as 100%. Data are means ± standard deviation of three independent digestions. Different letters above the bars indicate significant differences at *p* < 0.05 (two-way ANOVA followed by Duncan’s Multiple Range Test). D3G, delphinidin-3-*O*-glucoside; C3G, cyanidin-3-*O*-glucoside; Pet3G, petunidin-3-*O*-glucoside; Peo3G, peonidin-3-*O*-glucoside; M3G, malvidin-3-*O*-glucoside; Peo3AG, peonidin-3-*O*-acetylglucoside; C3CG, cyanidin-3-*O*-*p*-coumaroylglucoside; Pet3CG, petunidin-3-*O*-*p*-coumaroylglucoside.

For individual anthocyanins, significant effects were predominantly associated with α-casein. In Fetească Neagră, gastric recovery increased significantly for petunidin-3-*O*-glucoside, while the intestinal-phase improvements concerned mainly the major anthocyanins (Figure 4). The same pattern was found in Merlot, the largest gastric increases being recorded for delphinidin-3-*O*-glucoside and malvidin-3-*O*-glucoside (Figure 5). In Pinot Noir, significant effects were observed for both caseins and in both phases (Figure 6). Petunidin-3-*O*-*p*-coumaroylglucoside in Merlot was the only compound for which β-casein produced significantly higher digestive recovery than that of α-casein.

### 3.8. Relationships Between Phytochemical Composition and Antioxidant Capacity

**Figure 7:**
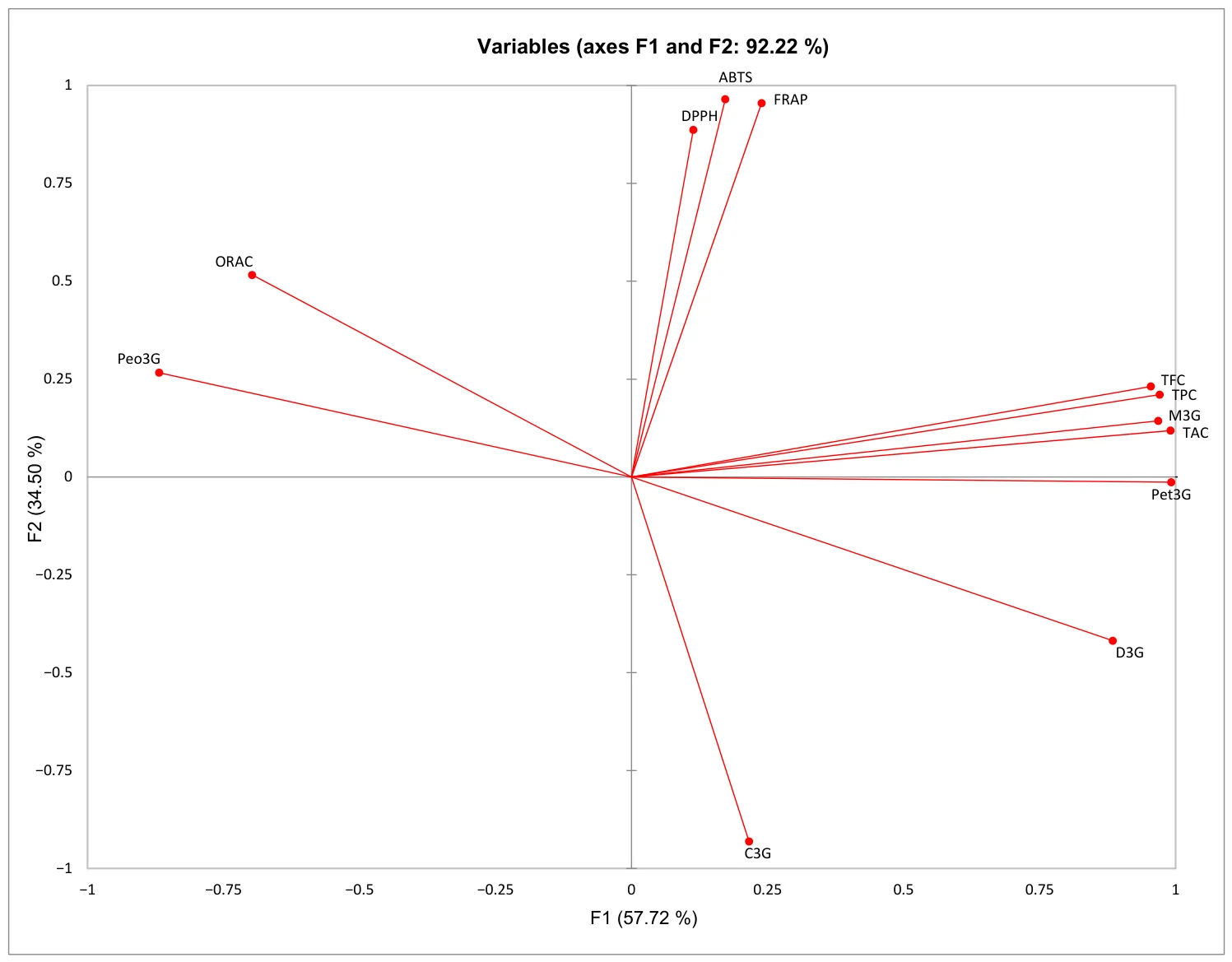
Principal component analysis (PCA) of the phytochemical composition, antioxidant activity and major anthocyanins in red grape (*Vitis vinifera* L.) cultivars. TAC, total anthocyanin content; TPC, total polyphenol content; TFC, total flavonoid content; DPPH, 2,2-diphenyl-1-picrylhydrazyl assay; ABTS, 2,2′-azino-bis(3-ethylbenzothiazoline-6-sulfonic acid) assay; ORAC, oxygen radical absorbance capacity assay; FRAP, ferric reducing antioxidant power assay; D3G, delphinidin-3-*O*-glucoside; C3G, cyanidin-3-*O*-glucoside; Pet3G, petunidin-3-*O*-glucoside; Peo3G, peonidin-3-*O*-glucoside; M3G, malvidin-3-*O*-glucoside.

The PCA was used to summarize the compositional and antioxidant profile of each cultivar and to identify which measured variables move together. The biplot (Figure 7) of the phytochemical composition, antioxidant capacity, and major anthocyanins of the three red grape (*Vitis vinifera* L.) cultivars shows that the first two principal components, F1 and F2, explain 92.22% of the total variance (F1, 57.72%; F2, 34.50%). F1 primarily separates cultivars based on total phenolic and anthocyanin content. Correlation patterns are also visible where closely aligned vectors indicate strong positive correlations, as seen between TAC, TPC, M3G, Pet3G, and TFC, as well as between antioxidant assays (DPPH, FRAP, ABTS). Conversely, vectors in opposite directions, such as C3G relative to ORAC, suggest negative correlations.

### 3.9. Main Effects of Cultivar and Casein Treatment on the Recovery of Major Anthocyanins

The main effects of cultivar and casein treatment on the recovery of the major anthocyanins were assessed by two-way ANOVA (Table S2). In the gastric phase, statistical analysis revealed a significant effect of cultivar on the digestive recovery of cyanidin-3-*O*-glucoside (F(2,12) = 7.173, *p* = 0.0089) and petunidin-3-*O*-glucoside (F(2,12) = 8.363, *p* = 0.0053). Additionally, treatment with casein significantly affected the digestive recovery of the major anthocyanins, except for cyanidin-3-*O*-glucoside. Moreover, no significant interactions between cultivar and casein treatment were detected for any of the anthocyanins in the gastric phase. On the other hand, in the intestinal phase, the analysis of variance showed significant main effects of both cultivar and casein treatment on the digestive recovery of all major anthocyanins. A significant interaction between cultivar and casein treatment was observed only for the digestive recovery of delphinidin-3-*O*-glucoside.

## 4. Discussion

The pH and titratable acidity of the three cultivars (3.30–3.60 and 4.30–6.20 g L^−1^, respectively) did not differ significantly (Table 1) and are consistent with values reported for *V. vinifera* berries at technological maturity in temperate viticultural areas [69]. Compared with data for Fetească Neagră and Pinot Noir grown in the Cluj and Mica areas of Transylvania [70], where Pinot Noir showed the highest pH and the lowest acidity, the present ranking is reversed. This most likely reflects the 2024 growing season, marked by high temperatures and uneven precipitation, conditions that accelerate malic acid respiration and raise berry pH, together with differences in vineyard site, crop load and harvest date [71]; seasonal and site effects therefore appear to outweigh the varietal contribution to these parameters. All three cultivars nevertheless fell within a narrow acidic window in which anthocyanins are relatively stable, so that pH-driven degradation is expected to occur during digestion rather than within the berry.

Anthocyanins contribute to antioxidant activity, although the strength of the correlation varies with the assay and with the contribution of other phenolic compounds [72,73,74]. In the present study, Fetească Neagră showed the highest polyphenol and flavonoid contents and the strongest activity in the DPPH, ABTS and FRAP assays, with significant differences for ABTS and FRAP, whereas no significant differences were found for ORAC, in which Merlot recorded the highest value. The total phenolic contents obtained here (286.92–535.01 mg GAE/100 g FW) fall within the range of 95.3–686.5 mg/100 g reported for twenty-four *V. vinifera* cultivars [75] and are comparable to the value reported by Yang et al. for Pinot Noir (396.8 mg GAE/100 g FW) [76], but markedly higher than those of Costa et al. for Pinot Noir and Merlot (38.6 and 37.7 mg GAE/100 g FW) [77]. Such divergence is attributable mainly to climate, followed by soil type, cultivar and water availability [51]. Since the measured antioxidant capacity depends on the reaction mechanism involved, on solvent polarity and on extraction conditions, values should only be compared across studies using the same method [78]. Applying the weighted antioxidant index [79] to the DPPH, ABTS and ORAC results, with FRAP being expressed as mmol Fe^2+^/g, confirmed Fetească Neagră as the cultivar with the highest overall antioxidant capacity (Table S3). To our knowledge, no previous study has evaluated the antioxidant activity of grape skin extracts obtained using acidified methanol from these three grape cultivars.

The qualitative and quantitative anthocyanin composition is a key determinant of stability during digestion, since different structures respond differently to gastrointestinal conditions. All three cultivars contained the five monoglucosides characteristic of red grapes [80], and the elution order matched that reported for *V. vinifera* anthocyanins on reversed-phase C18 columns [81], with malvidin-3-*O*-glucoside predominating in all samples, in agreement with previous reports [15,77,82,83,84]. Pinot Noir showed the smallest acylated fraction and no detectable malvidin-3-*O*-*p*-coumaroylglucoside, consistent with reports describing it as the cultivar with the lowest degree of acylation [85,86,87]. Unlike studies reporting a complete absence of acylated derivatives, small amounts of peonidin-3-*O*-acetylglucoside and *p*-coumaroylated forms were quantified here, a difference plausibly related to clone, rootstock and vine vigour [86]. In Merlot, *p*-coumaroylated derivatives predominate over acetylated ones, in contrast to reports from other growing regions [87,88], with the acetyl-to-coumaroyl ratio being strongly modulated by region, season and ripening stage. Anthocyanin composition is therefore shaped by genotype and environment alike, with implications for stability and bioactivity [89,90,91].

Anticancer effects of grape extracts have been studied mainly in seeds and attributed to epigallocatechin and procyanidins [92,93], although constituents of other berry fractions are also likely to contribute [10,94]. Our objective was therefore to evaluate the effect on cell viability of an anthocyanin-rich extract obtained from grape skins, using Fetească Neagră, the cultivar richest in anthocyanins. The extract reduced melanoma cell viability in a concentration-dependent manner, with no effect at 0.4 µg/mL. Comparable effects have been reported on the same cell line: a 30% reduction in viability after 48 h with a strawberry anthocyanin-rich extract at 333 µg/g FW [95], and inhibition of proliferation together with induction of apoptosis by blueberry extracts at 50–750 µg/mL [96,97]. Both the anthocyanin profiles and the concentrations used in those studies differ substantially from ours, which underlines the need to establish how specific profiles influence cancer cell behaviour, particularly since synergism between polyphenols has been proposed [98]. Polyphenols act through metabolic, cellular, and molecular mechanisms regulating gene expression, signalling pathways, and epigenetic changes [99,100], the main anti-melanoma activities of natural compounds being the potentiation of apoptosis, the reduction in cell growth and the prevention of metastasis [101].

Since the physiological relevance of dietary anthocyanins depends on the fraction that survives digestion in soluble form, anthocyanin recovery during digestion was evaluated using a simulated gastrointestinal model, previous work having shown that pH changes, enzymatic activity and interactions with other food components alter both the stability and the recovery during digestion of these compounds [102,103,104]. Overall, anthocyanin digestive recovery was considerably higher after the gastric phase (~43–46%) compared with the intestinal phase (~10–12%). These findings are consistent with previous studies on anthocyanins from other grape cultivars where 45% of anthocyanins from Syrah grapes were reported bioaccessible after the gastric phase [28], and 7.6% of anthocyanins were bioaccessible after the intestinal phase [29]. This decrease is attributable to the transition from the acidic gastric environment to near-neutral intestinal pH: the flavylium cation predominates under acidic conditions, whereas at neutral pH, hydration, structural transformation and oxidation reduce the intact fraction. Under neutral or slightly basic salivary conditions, up to 30% of cyanidin-3-*O*-glucoside may convert to the chalcone form [105].

Considering the well-recognised interactions between dietary proteins and polyphenols, caseins are likely to modulate anthocyanin behaviour during digestion by forming protein–polyphenol complexes [106], an interaction that can occur under physiological conditions since anthocyanin-rich foods are frequently consumed alongside dairy products. The experimental finding of the present study is that α-casein increased the recovery of anthocyanins in both digestive phases and in all three cultivars, whereas β-casein did so only in Pinot Noir and only after the intestinal phase. This contrasts with observations on blueberries, where α- or β-casein had little effect during the oral phase and a slight decrease in stability was recorded during gastric digestion [39]. The molecular basis of this difference was not investigated here, and the following interpretation is therefore offered as a hypothesis derived from published work. Caseins are intrinsically disordered, rheomorphic proteins with an open conformation and a high proportion of exposed hydrophobic surface [43]. He et al. reported that both α- and β-casein interact with malvidin-3-*O*-glucoside and improve the stability of grape skin anthocyanin extracts, with a higher binding affinity for α-casein [46], while Yin et al. showed that casein-derived hydrolysates retain the capacity to bind cyanidin-3-*O*-glucoside [48]. In addition, Lang et al. observed a lower susceptibility of α-casein to enzymatic digestion compared with β-casein [39]. In our study, the effects of casein addition on individual anthocyanins were most pronounced for the predominant compounds, particularly the non-acylated 3-glucosides: in Fetească Neagră, a significant increase was recorded for petunidin-3-*O*-glucoside during the gastric phase and for the major anthocyanins during the intestinal phase, while in Merlot the largest gastric increases concerned delphinidin-3-*O*-glucoside and malvidin-3-*O*-glucoside. Taken together, these published observations would account for the pattern reported here, but direct evidence is required before any binding mechanism can be attributed to the effect observed in this study.

The effect of casein addition was not uniform across the individual anthocyanins, which suggests that binding depends on the structure of the aglycone and on the presence of acyl substituents. The B-ring substitution pattern determines the balance between the interaction modes available: anthocyanins bearing free hydroxyl groups, such as delphinidin and petunidin derivatives, provide hydrogen-bond donors and have been reported to interact with proteins mainly through enthalpically favourable contributions, whereas the fully methoxylated malvidin interacts predominantly through entropically driven hydrophobic contacts [107]. In studies on anthocyanidin–protein binding, affinity decreased with increasing methoxylation [108], although the same work found the pattern to be irregular for the corresponding glucosides, indicating that the sugar moiety modulates the structure–affinity relationship. Acylation adds a further dimension, since aromatic acyl groups attached to the sugar fold back over the chromophore and form a conformation that shields the reactive positions [109]: such an intramolecular arrangement is expected to reduce the surface available for protein binding, so that acylated anthocyanins would rely more on intramolecular stabilisation and less on protein binding. This combination of opposing contributions is consistent with the compound-specific pattern observed here, in particular with petunidin-3-*O*-*p*-coumaroylglucoside in Merlot being the only anthocyanin for which β-casein was more effective than α-casein.

Being readily accessible to gastric and pancreatic proteases, caseins are extensively hydrolysed during digestion, and the species present at the end of the intestinal phase are peptides rather than intact proteins. The protection observed here can therefore originate from three successive contributions: binding to the intact protein during the oral and early gastric phases, binding to partially hydrolysed intermediates, and interaction with casein-derived peptides in the intestinal phase [48]. β-Casein is a preferred pepsin substrate, its hydrophobic C-terminal domain being cleaved early during gastric digestion, whereas α-s1-casein has been reported to be comparatively more resistant under the same conditions [39]. A faster loss of the binding-competent structure of β-casein would be consistent with the limited and cultivar-dependent effect recorded here for this protein. As the degree of hydrolysis and the size distribution of the resulting assemblies were not determined, this interpretation remains a hypothesis.

The cultivar-specific anthocyanin composition offers a plausible explanation for the varietal patterns observed during digestion. Beyond limiting protein binding, as discussed above, acylation stabilizes the flavylium chromophore through intramolecular copigmentation, which restricts the hydration reaction predominating at near-neutral pH [109]. Consistently, the acylated derivatives showed the lowest digestive recovery in the gastric phase, where release from the matrix rather than chemical stability is limiting, but were among the best retained after the intestinal phase, with delphinidin-3-*O*-*p*-coumaroylglucoside and petunidin-3-*O*-acetylglucoside recording the highest intestinal values in Fetească Neagră. The comparatively high acylated fraction of Fetească Neagră (19.5%) is therefore consistent with its higher intestinal recovery of total anthocyanins, whereas the predominantly monoglucosidic profile of Pinot Noir (92.4%) leaves its pigments more exposed to pH-driven degradation. The simpler anthocyanin profile of Pinot Noir may equally account for the fact that this was the only cultivar in which β-casein significantly improved digestive recovery, since fewer competing structures were available for interaction with the protein.

The two experimental lines address different questions and were not designed to be combined quantitatively: the digestion experiments indicate what proportion of the anthocyanins remains in solution, while the cell assay indicates the concentration range in which an effect becomes measurable. Establishing whether the amounts recovered after digestion fall within that range would require the cell assay to be performed directly on the intestinal digesta. Extending this work to a cellular environment would also require accounting for conditions absent from the digestion model: at 37 °C the self-association of β-casein would be favoured, potentially sequestering bound anthocyanins within aggregates; culture media buffered near pH 7.4 present anthocyanins predominantly as colourless carbinol and chalcone forms, which protein binding may partially counteract; and the thirteen anthocyanins of the extract would compete for a limited number of binding sites, so that the free fraction of each compound would depend on its relative abundance as much as on its intrinsic affinity. Confirming the present findings and addressing these questions would require digestion of the intact grape matrix, characterization of the complexes by fluorescence quenching, particle size and ζ-potential measurements, quantification of the degree of hydrolysis, Caco-2 transport experiments, and cell assays performed on the intestinal digesta with and without caseins, together with casein-only controls, before proceeding to animal models or clinical studies.

Several limitations should be considered when interpreting these results. The digestion substrate was a crude acidified methanolic extract, obtained with a solvent that is not food-grade, rather than intact grape skin, so the reported values describe the digestive stability and solubility of pre-extracted anthocyanins and represent an upper estimate of what would be obtained from whole grapes; a formal mass balance was not established, as the pellet fraction was not analysed, and the non-recovered fraction therefore comprises jointly degraded, insolubilized and possibly protein-co-precipitated anthocyanins. A single protein concentration was tested; the degree of casein hydrolysis and the size of the assemblies formed were not monitored, and the commercial α-casein preparation was a mixture of αs1- and αs2-casein (≥70%), whereas β-casein was a highly purified single protein, so the effects attributed to α-casein reflect the behaviour of that fraction as a whole. The digestion model lacks the gut microbiota, no cellular absorption model was coupled to the intestinal digesta, and the cell viability assay was deliberately performed with the undigested anthocyanin-rich extract rather than with the digesta: since the digested samples contain caseins, their hydrolysis products, digestive enzymes and bile salts, any effect observed on the cells could not have been attributed unambiguously to the anthocyanins, and the assay was therefore designed to characterise the extract itself.

## 5. Conclusions

This study shows that milk caseins protect grape anthocyanins during simulated gastrointestinal digestion, and that the magnitude of this effect depends on both the casein fraction and the cultivar. α-Casein increased anthocyanin recovery in all three cultivars and in both digestive phases, whereas β-casein had a more limited and cultivar-dependent effect. The initial hypothesis was therefore supported for α-casein but only partially for β-casein. Anthocyanin recovery during digestion was markedly higher after the gastric phase than after the intestinal phase, consistent with the pH-driven instability of anthocyanins at near-neutral pH, and casein addition partially counteracted this loss. Since the interaction itself was not measured, the mechanisms proposed here remain hypotheses derived from published work rather than experimentally demonstrated properties of the systems studied.

The three cultivars differed in phytochemical composition, malvidin-3-*O*-glucoside being the major anthocyanin in all of them, while the individual and acylated anthocyanins varied; Fetească Neagră showed the highest polyphenol and flavonoid content and the strongest antioxidant activity. The extract of this cultivar reduced the viability of B16-F10 murine melanoma cells in a concentration-dependent manner.

These findings indicate that the digestive behaviour of grape anthocyanins depends on cultivar-specific composition and on interactions with dietary proteins, and that casein–anthocyanin systems deserve further investigation for functional food applications. Confirmation would require digestion of the intact grape matrix, direct characterization of the complexes formed and, ultimately, in vivo studies.

## Figures and Tables

**Table 1:** Physicochemical composition of red grapes (*V. vinifera* L.).

Grape (*V. vinifera* L.) Cultivars	pH	Titratable Acidity (g L^−1^)
Fetească Neagră	3.60 ± 0.37 ^a^	5.90 ± 0.33 ^a^
Merlot	3.30 ± 0.45 ^a^	4.30 ± 0.66 ^a^
Pinot Noir	3.43 ± 0.61 ^a^	6.20 ± 0.24 ^a^

Data expressed as mean values ± standard deviation (measurements carried out in triplicate). In columns, mean values with different superscript letters denote significant differences at *p* < 0.05 (Duncan’s Multiple Range Test).

**Table 2:** Total anthocyanin, total polyphenol, and total flavonoid content in red grapes (*V. vinifera* L.).

Grape (*V. vinifera* L.) Cultivars	Total Anthocyanins (mg MGE/100 g FW)	Total Polyphenols (mg GAE/100 g FW)	Total Flavonoids (mg CE/100 g FW)
Fetească Neagră	220.36 ± 9.04 ^a^	535.01 ± 38.43 ^a^	202.52 ± 30.61 ^a^
Merlot	189.33 ± 8.83 ^b^	412.33 ± 20.94 ^b^	126.41 ± 12.14 ^b^
Pinot Noir	134.21 ± 12.58 ^c^	286.92 ± 29.35 ^c^	57.48 ± 22.53 ^c^

Data expressed as mean values ± standard deviation (measurements carried out in triplicate). In columns, mean values with different superscript letters denote a significant difference at *p* < 0.05 (Duncan’s Multiple Range Test). Abbreviations: MGE, malvidin-3-*O*-glucoside equivalents; GAE, gallic acid equivalents; CE, catechin equivalents; FW, fresh weight.

**Table 3:** Comparison of antioxidant activity in red grapes (*V. vinifera* L.).

Grape (*V. vinifera* L.) Cultivars	DPPH (mmol TE/g)	ABTS (mmol TE/g)	ORAC (mmol TE/g)	FRAP (mmol Fe^2+^/g)
Fetească Neagră	2.12 ± 0.15 ^a^	2.11 ± 0.08 ^a^	3.51 ± 0.27 ^a^	15.57 ± 0.14 ^a^
Merlot	1.93 ± 0.16 ^ab^	1.79 ± 0.02 ^b^	4.01 ± 0.38 ^a^	12.49 ± 0.53 ^b^
Pinot Noir	1.72 ± 0.21 ^b^	1.33 ± 0.02 ^c^	3.35 ± 0.34 ^a^	9.04 ± 0.22 ^c^

Data expressed as mean values ± standard deviation (measurements carried out in triplicate). In columns, mean values with different superscript letters denote significant difference at *p* < 0.05 (Duncan’s Multiple Range Test). Abbreviations: DPPH, 2,2-diphenyl-1-picrylhydrazyl radical scavenging assay; ABTS, 2,2′-azino-bis(3-ethylbenzothiazoline-6-sulfonic acid) radical cation scavenging assay; ORAC, oxygen radical absorbance capacity assay; FRAP, ferric reducing antioxidant power assay; TE, Trolox equivalents; Fe^2+^, ferrous ion equivalents.

**Table 4:** Retention times, UV-VIS and mass spectral data of anthocyanins identified in red grapes (*V. vinifera* L.).

Peak No.	Rt (min)	UV λ_max_ (nm)	[M+H]+ (*m/z*)	Compound
An1	22.03	280, 523	465	Delphinidin-3-*O*-glucoside
An2	25.85	279, 515	449	Cyanidin-3-*O*-glucoside
An3	28.62	277, 526	479	Petunidin-3-*O*-glucoside
An4	31.77	279, 515	463	Peonidin-3-*O*-glucoside
An5	33.23	278, 530	493	Malvidin-3-*O*-glucoside
An6	37.82	271, 530	521	Petunidin-3-*O*-acetylglucoside
An7	38.41	278, 530	611	Delphinidin-3-*O*-*p*-coumaroylglucoside
An8	39.28	280, 518	505	Peonidin-3-*O*-acetylglucoside
An9	42.49	280, 521	535	Malvidin-3-*O*-acetylglucoside
An10	43.19	271, 522	595	Cyanidin-3-*O-p*-coumaroylglucoside
An11	43.94	276, 531	625	Petunidin-3-*O*-*p*-coumaroylglucoside
An12	45.34	279, 523	609	Peonidin-3-*O*-*p*-coumaroylglucoside
An13	47.99	280, 521	639	Malvidin-3-*O*-*p*-coumaroylglucoside

**Table 5:** Individual anthocyanin content in red grapes (*V. vinifera* L.), expressed as mg MGE/100 g FW.

Peak No.	Anthocyanin Compound	Grape (*V. vinifera* L.) Cultivars		
		Fetească Neagră	Merlot	Pinot Noir
An1	Delphinidin-3-*O*-glucoside	12.94 ± 0.11 ^b^	14.80 ± 0.81 ^a^	5.06 ± 0.25 ^c^
An2	Cyanidin-3-*O*-glucoside	3.24 ± 0.16 ^b^	7.55 ± 1.48 ^a^	3.45 ± 0.50 ^b^
An3	Petunidin-3-*O*-glucoside	18.38 ± 0.66 ^a^	15.65 ± 0.33 ^b^	7.80 ± 0.17 ^c^
An4	Peonidin-3-*O*-glucoside	18.85 ± 1.39 ^b^	17.47 ± 2.29 ^b^	35.71 ± 2.57 ^a^
An5	Malvidin-3-*O*-glucoside	124.07 ± 1.94 ^a^	101.18 ± 4.62 ^b^	71.96 ± 14.23 ^c^
An6	Petunidin-3-*O*-acetylglucoside	1.12 ± 0.14 ^a^	0.91 ± 0.06 ^b^	nd
An7	Delphinidin-3-*O*-*p*-coumaroylglucoside	1.08 ± 0.05 ^a^	nd	nd
An8	Peonidin-3-*O*-acetylglucoside	2.77 ± 0.36 ^b^	4.26 ± 0.62 ^ab^	6.63 ± 2.75 ^a^
An9	Malvidin-3-*O*-acetylglucoside	2.22 ± 0.16 ^a^	2.37 ± 0.09 ^a^	nd
An10	Cyanidin-3-*O-p*-coumaroylglucoside	1.76 ± 0.06 ^b^	2.51 ± 0.33 ^a^	1.62 ± 0.15 ^b^
An11	Petunidin-3-*O*-*p*-coumaroylglucoside	3.45 ± 0.26 ^a^	2.50 ± 0.39 ^b^	1.99 ± 0.07 ^b^
An12	Peonidin-3-*O*-*p*-coumaroylglucoside	2.90 ± 0.20 ^a^	2.58 ± 0.20 ^b^	nd
An13	Malvidin-3-*O*-*p*-coumaroylglucoside	27.58 ± 3.55 ^a^	17.57 ± 0.09 ^b^	nd
	Σ Monoglucosides	177.48 ± 4.26 (80.5%)	156.65 ± 9.53 (82.7%)	123.98 ± 17.72 (92.4%)
	Σ Acetylglucosides	6.11 ± 0.66 (2.8%)	7.54 ± 0.77 (4.0%)	6.63 ± 2.75 (4.9%)
	Σ *p*-coumaroylglucosides	36.77 ± 4.12 (16.7%)	25.16 ± 1.01 (13.3%)	3.61 ± 0.22 (2.7%)
	Σ Acylated anthocyanins	42.88 ± 4.78 (19.5%)	32.70 ± 1.78 (17.3%)	10.24 ± 2.97 (7.6%)

nd—not detected. Data expressed as mean ± standard deviation (measurements carried out in triplicate). In rows, mean values with different superscript letters denote significant differences at *p* < 0.05 (Duncan’s Multiple Range Test). Values in parentheses in the summary rows represent the percentage of each anthocyanin class relative to the sum of individual anthocyanins. Monoglucosides comprise An1–An5; acetylglucosides comprise An6, An8, and An9; *p*-coumaroylglucosides comprise An7 and An10–An13.

## Data Availability

The original contributions presented in this study are included in the article/Supplementary Materials. Further inquiries can be directed to the corresponding author.

## Supplementary Materials

The following supporting information can be downloaded at https://www.mdpi.com/article/10.3390/foods15183295/s1: Figure S1: HPLC chromatogram of the anthocyanin-rich fraction of the Fetească Neagră cultivar; Figure S2: HPLC chromatogram of the anthocyanin-rich fraction of the Merlot cultivar; Figure S3: HPLC chromatogram of the anthocyanin-rich fraction of the Pinot Noir cultivar; Figure S4: Morphology of non-treated B16-F10 murine melanoma cells, scale bar = 20 µm; Figure S5: Morphology of B16-F10 murine melanoma cells treated with 10 µg malvidin-3-O-glucoside equivalents/mL extract of Fetească Neagră cv., scale bar = 20 µm; Table S1: Individual anthocyanin recovery during digestion in red grape (V. vinifera L.) cultivars, expressed as percentage of initial grape extract (100%); Table S2: Two-way ANOVA results for the effect of casein treatment and cultivar on the digestive recovery of major anthocyanins from red grapes (V. vinifera L.) during gastric and intestinal digestion phases; Table S3: Standardized antioxidant capacities of red grape (V. vinifera L.) cultivars with respect to Trolox measurements.
