# Supplementary material for: α- and β-Casein Differentially Protect Anthocyanins from Three Red Grape (Vitis vinifera L.) Cultivars During Simulated Gastrointestinal Digestion
Source: Foods. 2026 Sep 17;15(18):3295. doi: 10.3390/foods15183295 (PMC13606855; doi:10.3390/foods15183295)
Supplement: Supplementary file 1 [file foods-15-03295-s001.zip › foods-4517644-supplementary.pdf]

Table S1. Individual anthocyanin recovery during digestion in red grape (*V. vinifera* L.) cultivars, expressed as percentage of initial grape extract (100%).

**Fetească Neagră**						
	An (G)	An + α-casein (G)	An + β-casein (G)	An (I)	An + α-casein (I)	An + β-casein (I)
Anthocyanin compound	%	%	%	%	%	%
D3G	30.06 ± 3.37 ^{a}	37.07 ± 6.31 ^{a}	28.02 ± 4.40 ^{a}	2.20 ± 0.35 ^{a}	7.76 ± 1.35 ^{a}	3.42 ± 0.49 ^{a}
C3G	38.60 ± 3.89 ^{a}	42.60 ± 6.29 ^{a}	33.99 ± 4.20 ^{a}	10.67 ± 1.50 ^{b}	21.97 ± 3.22 ^{a}	14.86 ± 2.65 ^{b}
Pet3G	39.26 ± 3.75 ^{b}	49.19 ± 4.54 ^{a}	38.50 ± 5.88 ^{b}	7.35 ± 1.78 ^{b}	25.18 ± 5.49 ^{a}	13.95 ± 4.13 ^{b}
Peo3G	62.41 ± 7.93 ^{a}	62.28 ± 10.27 ^{a}	47.89 ± 6.46 ^{a}	14.83 ± 2.82 ^{b}	33.14 ± 4.93 ^{a}	21.91 ± 5.71 ^{b}
M3G	49.68 ± 3.82 ^{a}	61.64 ± 7.82 ^{a}	50.00 ± 5.03 ^{a}	16.27 ± 2.84 ^{b}	35.82 ± 5.47 ^{a}	23.88 ± 6.85 ^{b}
Pet3AG	32.35 ± 13.18 ^{a}	15.24 ± 0.37 ^{a}	23.35 ± 6.83 ^{a}	15.29 ± 2.04 ^{a}	14.38 ± 0.40 ^{a}	16.15 ± 2.02 ^{a}
D3CG	23.39 ± 4.04 ^{a}	21.91 ± 1.89 ^{a}	27.18 ± 7.40 ^{a}	17.44 ± 1.37 ^{a}	17.09 ± 0.84 ^{a}	19.27 ± 2.24 ^{a}
Peo3AG	44.30 ± 9.29 ^{a}	32.81 ± 0.49 ^{ab}	30.96 ± 4.94 ^{b}	8.30 ± 0.82 ^{b}	10.71 ± 1.37 ^{a}	12.29 ± 0.64 ^{a}
M3AG	28.80 ± 8.00 ^{a}	29.20 ± 5.95 ^{a}	15.50 ± 10.60 ^{a}	7.03 ± 0.71 ^{a}	6.21 ± 0.47 ^{a}	6.07 ± 1.43 ^{a}
C3CG	27.21 ± 6.60 ^{a}	22.73 ± 6.65 ^{a}	24.29 ± 8.91 ^{a}	9.78 ± 0.91 ^{a}	13.35 ± 0.85 ^{a}	12.83 ± 4.72 ^{a}
Pet3CG	27.72 ± 9.42 ^{a}	14.70 ± 2.03 ^{ab}	16.21 ± 2.84 ^{b}	5.40 ± 0.45 ^{b}	9.10 ± 1.29 ^{ab}	11.87 ± 3.89 ^{a}
Peo3CG	30.49 ± 7.48 ^{a}	29.06 ± 5.85 ^{a}	21.41 ± 2.91 ^{a}	5.63 ± 0.60 ^{a}	5.04 ± 0.37 ^{ab}	4.49 ± 0.15 ^{b}
M3CG	19.27 ± 5.04 ^{a}	28.80 ± 9.77 ^{a}	20.97 ± 4.63 ^{a}	2.68 ± 0.19 ^{b}	4.25 ± 0.53 ^{a}	3.78 ± 0.59 ^{a}
**Merlot**						
	An (G)	An + α-casein (G)	An + β-casein (G)	An (I)	An + α-casein (I)	An + β-casein (I)
Anthocyanin compound	%	%	%	%	%	%
D3G	32.36 ± 4.24 ^{b}	43.02 ± 2.03 ^{a}	31.60 ± 0.61 ^{b}	1.68 ± 0.24 ^{c}	4.79 ± 0.18 ^{a}	2.11 ± 0.20 ^{b}
C3G	42.54 ± 5.28 ^{a}	49.55 ± 1.70 ^{a}	49.15 ± 8.92 ^{a}	8.59 ± 2.65 ^{b}	13.50 ± 0.74 ^{a}	7.94 ± 1.44 ^{b}
Pet3G	43.75 ± 7.17 ^{b}	56.00 ± 2.95 ^{a}	46.84 ± 2.39 ^{ab}	3.76 ± 0.51 ^{c}	11.40 ± 0.91 ^{a}	5.61 ± 2.05 ^{b}
Peo3G	60.66 ± 4.16 ^{a}	66.94 ± 0.85 ^{a}	58.07 ± 7.24 ^{a}	11.08 ± 0.87 ^{b}	18.11 ± 2.00 ^{a}	9.25 ± 2.58 ^{b}
M3G	53.64 ± 3.99 ^{b}	64.43 ± 6.87 ^{a}	53.65 ± 4.69 ^{b}	11.26 ± 0.72 ^{b}	20.17 ± 1.56 ^{a}	9.72 ± 2.73 ^{b}
Pet3AG	25.17 ± 6.18 ^{a}	30.21 ± 3.82 ^{a}	26.43 ± 10.09 ^{a}	14.81 ± 0.42 ^{a}	16.14 ± 1.31 ^{a}	13.79 ± 5.53 ^{a}
Peo3AG	33.39 ± 3.33 ^{a}	32.00 ± 1.94 ^{a}	44.95 ± 3.50 ^{a}	5.78 ± 3.05 ^{b}	12.40 ± 3.17 ^{a}	13.79 ± 3.65 ^{a}
M3AG	21.83 ± 4.48 ^{b}	35.78 ± 1.24 ^{a}	23.79 ± 7.24 ^{b}	7.29 ± 1.17 ^{b}	11.53 ± 1.68 ^{a}	6.92 ± 1.92 ^{b}
C3CG	17.21 ± 3.32 ^{a}	20.50 ± 2.98 ^{a}	16.30 ± 3.73 ^{a}	8.71 ± 2.99 ^{a}	11.29 ± 1.36 ^{a}	11.08 ± 5.01 ^{a}
Pet3CG	15.10 ± 6.67 ^{b}	14.27 ± 1.25 ^{b}	30.00 ± 6.87 ^{a}	8.48 ± 1.23 ^{a}	8.73 ± 0.84 ^{a}	6.92 ± 0.51 ^{a}
Peo3CG	22.30 ± 3.60 ^{b}	33.46 ± 2.96 ^{ab}	28.82 ± 6.38 ^{a}	6.81 ± 0.94 ^{a}	10.04 ± 1.08 ^{a}	8.36 ± 2.46 ^{a}
M3CG	24.62 ± 7.36 ^{a}	33.49 ± 1.58 ^{a}	26.72 ± 8.51 ^{a}	2.48 ± 1.14 ^{b}	6.34 ± 1.27 ^{a}	3.92 ± 0.76 ^{b}
**Pinot Noir**						
	An (G)	An + α-casein (G)	An + β-casein (G)	An (I)	An + α-casein (I)	An + β-casein (I)
Anthocyanin compound	%	%	%	%	%	%
D3G	30.48 ± 1.38 ^{b}	39.02 ± 3.27 ^{a}	30.57 ± 3.59 ^{b}	2.70 ± 0.70 ^{c}	4.82 ± 0.30 ^{a}	3.46 ± 0.46 ^{b}
C3G	40.66 ± 7.09 ^{a}	42.51 ± 5.70 ^{a}	34.96 ± 4.95 ^{a}	8.16 ± 1.25 ^{b}	15.16 ± 2.66 ^{a}	14.30 ± 0.71 ^{b}
Pet3G	39.84 ± 0.32 ^{b}	47.32 ± 2.56 ^{a}	37.25 ± 5.16 ^{ab}	4.92 ± 1.68 ^{c}	14.26 ± 4.45 ^{a}	14.13 ± 0.91 ^{b}
Peo3G	58.38 ± 10.87 ^{a}	63.98 ± 11.56 ^{a}	48.22 ± 7.72 ^{a}	10.92 ± 1.75 ^{b}	20.09 ± 2.96 ^{a}	19.56 ± 1.26 ^{b}
M3G	43.42 ± 0.95 ^{b}	57.01 ± 4.57 ^{a}	45.97 ± 4.50 ^{b}	11.92 ± 1.61 ^{b}	22.13 ± 4.02 ^{a}	22.18 ± 1.25 ^{b}

Peo3AG	25.67 ± 4.00 [a]	33.53 ± 4.53 [a]	16.57 ± 6.42 [a]	7.39 ± 0.92 [b]	11.73 ± 0.15 [a]	9.66 ± 0.46 [a]
C3CG	15.91 ± 3.36 [a]	21.66 ± 3.11 [a]	15.22 ± 2.69 [a]	13.67 ± 0.86 [b]	22.23 ± 4.00 [a]	17.17 ± 0.02 [a]
Pet3CG	16.22 ± 3.69 [b]	19.76 ± 4.72 [a]	14.36 ± 4.13 [b]	5.08 ± 0.77 [b]	11.17 ± 0.16 [a]	11.32 ± 1.57 [a]

Data expressed as mean ± standard deviation (measurements carried out in triplicate). In rows for gastric phase (G) and intestinal phase (I), mean values with different letters denote significant difference at $p < 0.05$ (Duncan's Multiple Range Test). D3G, delphinidin-3-*O*-glucoside; C3G, cyanidin-3-*O*-glucoside; Pet3G, petunidin-3-*O*-glucoside; Peo3G, peonidin-3-*O*-glucoside; M3G, malvidin-3-*O*-glucoside; Pet3AG, petunidin-3-*O*-acetylglucoside; D3CG, delphinidin-3-*O*-*p*-coumaroylglucoside; Peo3AG, peonidin-3-*O*-acetylglucoside; M3AG, malvidin-3-*O*-acetylglucoside; C3CG, cyanidin-3-*O*-*p*-coumaroylglucoside; Pet3CG, petunidin-3-*O*-*p*-coumaroylglucoside; Peo3CG, peonidin-3-*O*-*p*-coumaroylglucoside; M3CG, malvidin-3-*O*-*p*-coumaroylglucoside.

Table S2. Two-way ANOVA results for the effect of casein treatment and cultivar on the digestive recovery of major anthocyanins from red grapes (*V. vinifera* L.) during gastric and intestinal digestion phases.

**Delphinidin-3-*O*-glucoside – gastric phase**						
Anova Table	SS	DF	MS	F (DFn, DFd)	P value	P value summary
Interaction	7.452	2	3.726	F (2, 12) = 0.2562	P=0.7781	ns
Casein Treatment	418.5	1	418.5	F (1, 12) = 28.78	P=0.0002	***
Cultivar	68.33	2	34.17	F (2, 12) = 2.350	P=0.1377	ns
**Cyanidin-3-*O*-glucoside – gastric phase**						
Anova Table	SS	DF	MS	F (DFn, DFd)	P value	P value summary
Interaction	59.83	2	29.91	F (2, 12) = 0.9126	P=0.4276	ns
Casein Treatment	137.1	1	137.1	F (1, 12) = 4.184	P=0.0634	ns
Cultivar	470.2	2	235.1	F (2, 12) =7.173	P=0.0089	**
**Petunidin-3-*O*-glucoside – gastric phase**						
Anova Table	SS	DF	MS	F (DFn, DFd)	P value	P value summary
Interaction	1.777	2	0.8884	F (2, 12) = 0.05186	P=0.9497	ns
Casein Treatment	447.6	1	447.6	F (1, 12) = 26.13	P=0.0003	***
Cultivar	286.5	2	143.3	F (2, 12) = 8.363	P=0.0053	**
**Peonidin-3-3-*O*-glucoside – gastric phase**						
Anova Table	SS	DF	MS	F (DFn, DFd)	P value	P value summary
Interaction	39.91	2	19.95	F (2, 12) = 0.3042	P=0.7432	ns
Casein Treatment	761.3	1	761.3	F (1, 12) = 11.61	P=0.0052	**
Cultivar	194.2	2	97.11	F (2, 12) = 1.480	P=0.2663	ns
**Malvidin-3-*O*-glucoside – gastric phase**						
Anova Table	SS	DF	MS	F (DFn, DFd)	P value	P value summary
Interaction	0.5836	2	0.2918	F (2, 12) = 0.008897	P=0.9911	ns
Casein Treatment	559.8	1	559.8	F (1, 12) = 17.07	P=0.0014	**
Cultivar	172.2	2	86.12	F (2, 12) = 2.626	P=0.1133	ns
**Delphinidin-3-*O*-glucoside – intestinal phase**						
Anova Table	SS	DF	MS	F (DFn, DFd)	P value	P value summary
Interaction	6.689	2	3.345	F (2, 12) = 8.236	P=0.0056	**
Casein Treatment	35.11	1	35.11	F (1, 12) = 86.46	P<0.0001	****
Cultivar	14.32	2	7.158	F (2,12) = 17.63	P=0.0003	***
**Cyanidin-3-*O*-glucoside – intestinal phase**						
Anova Table	SS	DF	MS	F (DFn, DFd)	P value	P value summary
Interaction	31.78	2	15.89	F (2, 12) = 3.455	P=0.0653	ns
Casein Treatment	91.53	1	91.53	F (1, 12) = 19.90	P=0.0008	***
Cultivar	177.7	2	88.87	F (2, 12) = 19.33	P=0.0002	***
**Petunidin-3-*O*-glucoside – intestinal phase**						
Anova Table	SS	DF	MS	F (DFn, DFd)	P value	P value summary
Interaction	92.42	2	46.21	F (2, 12) = 3.805	P=0.0525	ns
Casein Treatment	147.1	1	147.1	F (1, 12) = 12.11	P=0.0045	**
Cultivar	367.1	2	183.5	F (2, 12) = 15.11	P=0.0005	***
**Peonidin-3-*O*-glucoside – intestinal phase**						
Anova Table	SS	DF	MS	F (DFn, DFd)	P value	P value summary
Interaction	94.75	2	47.37	F (2, 12) = 3.648	P=0.0578	ns

Casein Treatment	212.6	1	212.6	F (1, 12) = 16.37	P=0.0016	**
Cultivar	577.5	2	288.7	F (2, 12) = 22.23	P<0.0001	****
**Malvidin-3-*O*-glucoside – intestinal phase**						
Anova Table	SS	DF	MS	F (DFn, DFd)	P value	P value summary
Interaction	128.1	2	64.06	F (2, 12) = 3.680	P=0.0567	ns
Casein Treatment	249.5	1	249.5	F (1, 12) = 14.33	P=0.0026	**
Cultivar	666.7	2	333.4	F (2, 12)= 19.15	P=0.0002	***

SS = sum of squares; DF = degrees of freedom; MS = mean square; F = F-statistic. P values indicate statistical significance ($p < 0.05$). Significance levels are noted as: ns (not significant), * ($p < 0.05$), ** ($p < 0.01$), *** ($p < 0.001$), **** ($p < 0.0001$).

Table S3. Standardized antioxidant capacities of red grape (*V. vinifera* L.) cultivars with respect to Trolox measurements.

	**DPPH**	**ABTS**	**ORAC**	**Weighted average**
Fetească Neagră	2.12	2.11	3.51	*1.09*
Merlot	1.93	1.79	4.01	*1.05*
Pinot Noir	1.72	1.33	3.35	*0.86*
AVERAGE	1.92	1.74	3.62	

The right-hand column shows the weighted average (mmol TE/g) obtained by (1) dividing the antioxidant capacity of each cultivar, as determined by the specified method, by the average capacity determined for the whole set of compounds by the same method (bottom row), (2) summing the three (DPPH, ABTS and ORAC) results of this calculation, and (3) dividing the sum by three.

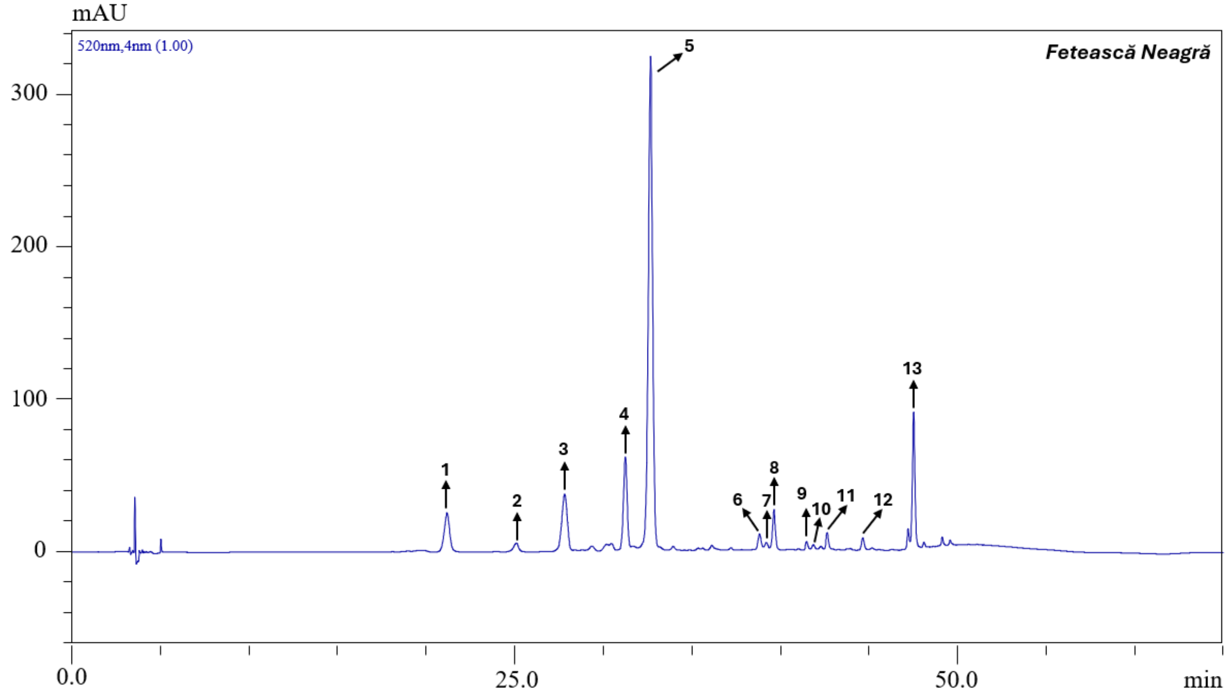

Figure S1. HPLC chromatogram of the anthocyanin-rich fraction of the Feteascӑ Neagrӑ cultivar. The HPLC chromatograms for anthocyanin separation in analyzed red grape cultivars (*V. vinifera* L.). (1) delphinidin-3-*O*-glucoside; (2) cyanidin-3-*O*-glucoside; (3) petunidin-3-*O*-glucoside; (4) peonidin-3-*O*-glucoside; (5) malvidin-3-*O*-glucoside; (6) petunidin-3-*O*-acetylglucoside; (7) delphinidin-3-*O*-*p*-coumaroylglucoside; (8) peonidin-3-*O*-acetylglucoside; (9) malvidin-3-*O*-acetylglucoside; (10) cyanidin-3-*O*-*p*-coumaroylglucoside; (11) petunidin-3-*O*-*p*-coumaroylglucoside; (12) peonidin-3-*O*-*p*-coumaroylglucoside; (13) malvidin-3-*O*-*p*-coumaroylglucoside (peak numbers correspond with those from Table 4). The identification of individual anthocyanins was made based on HPLC-ESI-MS.

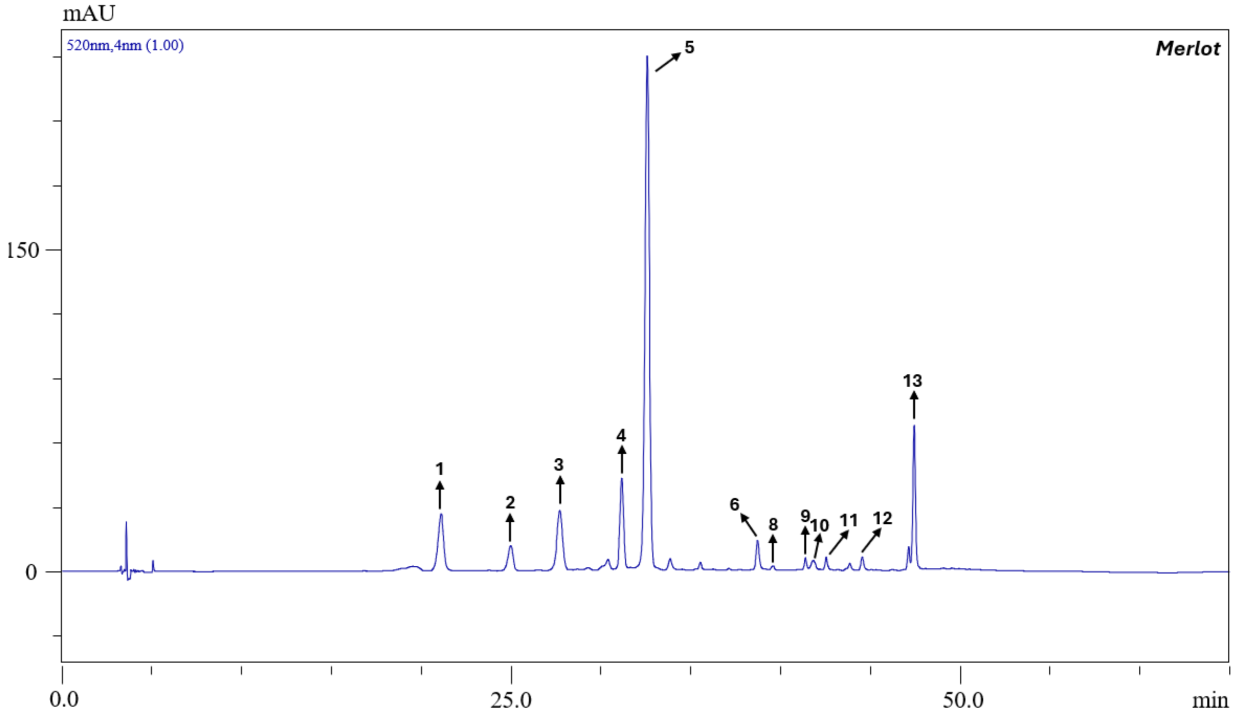

Figure S2. HPLC chromatogram of the anthocyanin-rich fraction of the Merlot cultivar. The HPLC chromatograms for anthocyanin separation in analyzed red grape cultivars (*V. vinifera* L.). (1) delphinidin-3-*O*-glucoside; (2) cyanidin-3-*O*-glucoside; (3) petunidin-3-*O*-glucoside; (4) peonidin-3-*O*-glucoside; (5) malvidin-3-*O*-glucoside; (6) petunidin-3-*O*-acetylglucoside; (8) peonidin-3-*O*-acetylglucoside; (9) malvidin-3-*O*-acetylglucoside; (10) cyanidin-3-*O*-*p*-coumaroylglucoside; (11) petunidin-3-*O*-*p*-coumaroylglucoside; (12) peonidin-3-*O*-*p*-coumaroylglucoside; (13) malvidin-3-*O*-*p*-coumaroylglucoside (peak numbers correspond with those from Table 4). The identification of individual anthocyanins was made based on HPLC-ESI-MS.

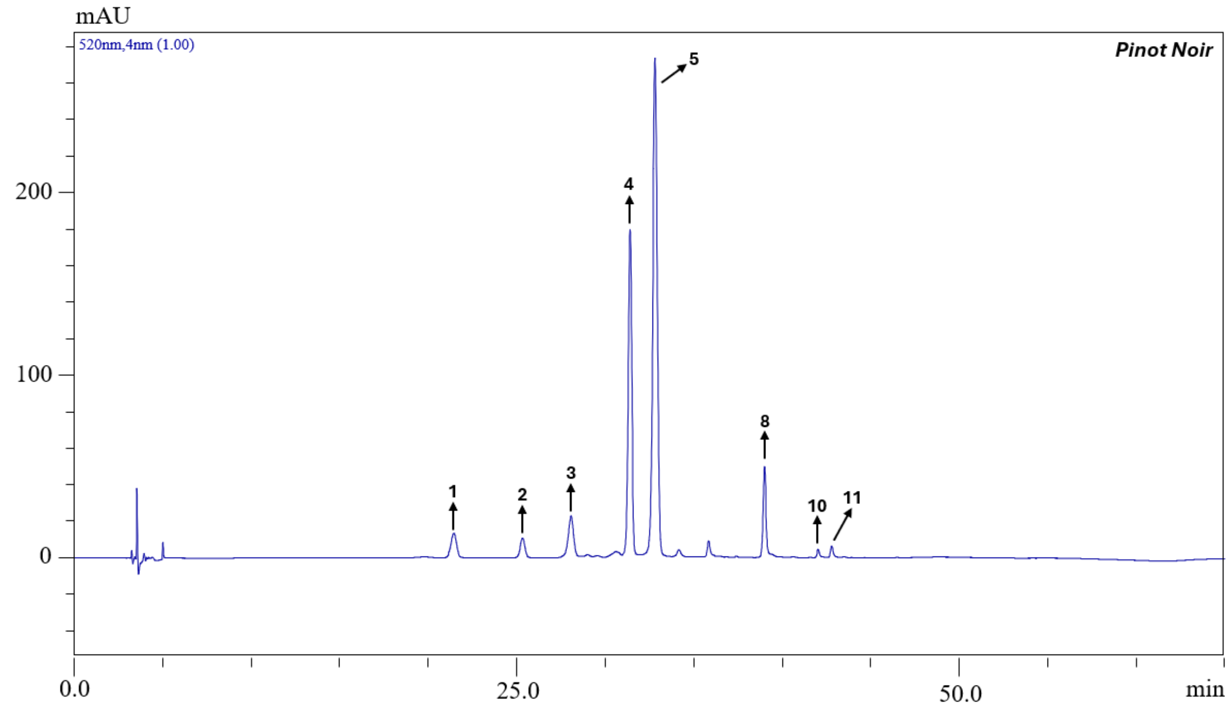

Figure S3. HPLC chromatogram of the anthocyanin-rich fraction of the Pinot Noir cultivar. The HPLC chromatograms for anthocyanin separation in analyzed red grape cultivars (*V. vinifera* L.). (1) delphinidin-3-*O*-glucoside; (2) cyanidin-3-*O*-glucoside; (3) petunidin-3-*O*-glucoside; (4) peonidin-3-*O*-glucoside; (5) malvidin-3-*O*-glucoside; (8) peonidin-3-*O*-acetylglucoside; (10) cyanidin-3-*O*-*p*-coumaroylglucoside; (11) petunidin-3-*O*-*p*-coumaroylglucoside (peak numbers correspond with those from Table 4). The identification of individual anthocyanins was made based on HPLC-ESI-MS.

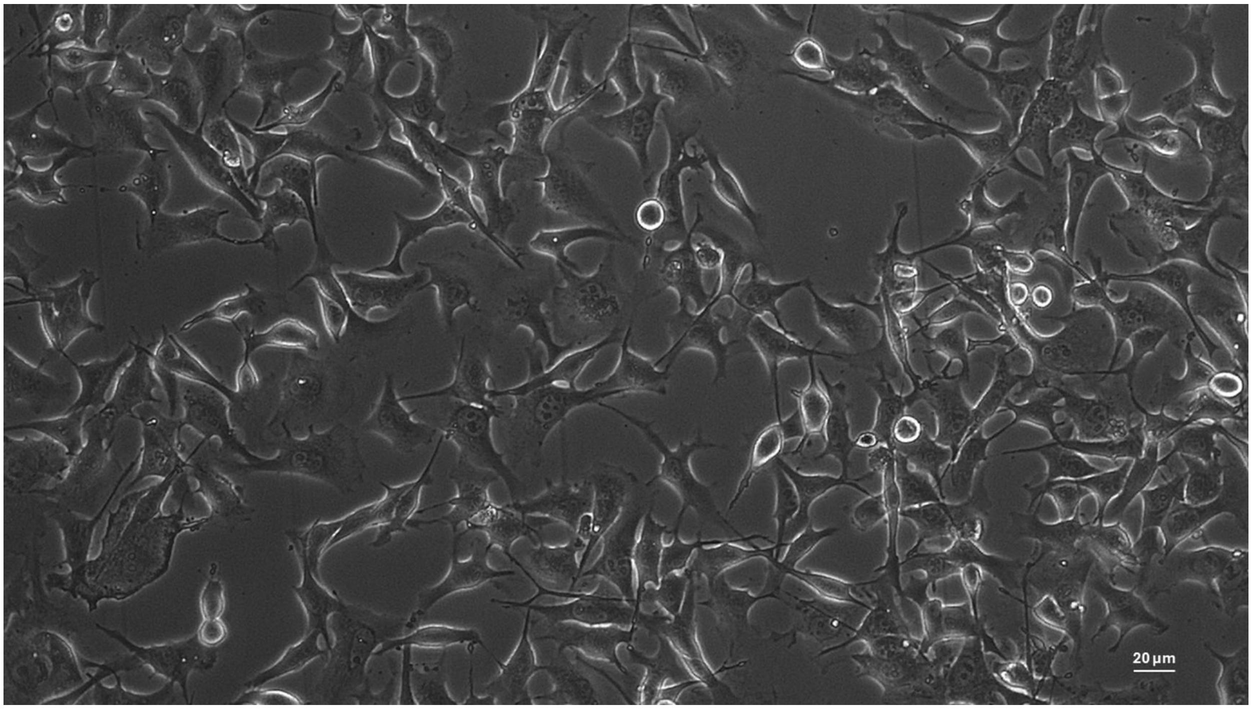

Figure S4. Morphology of non-treated B16-F10 murine melanoma cells, scale bar = 20 μm.

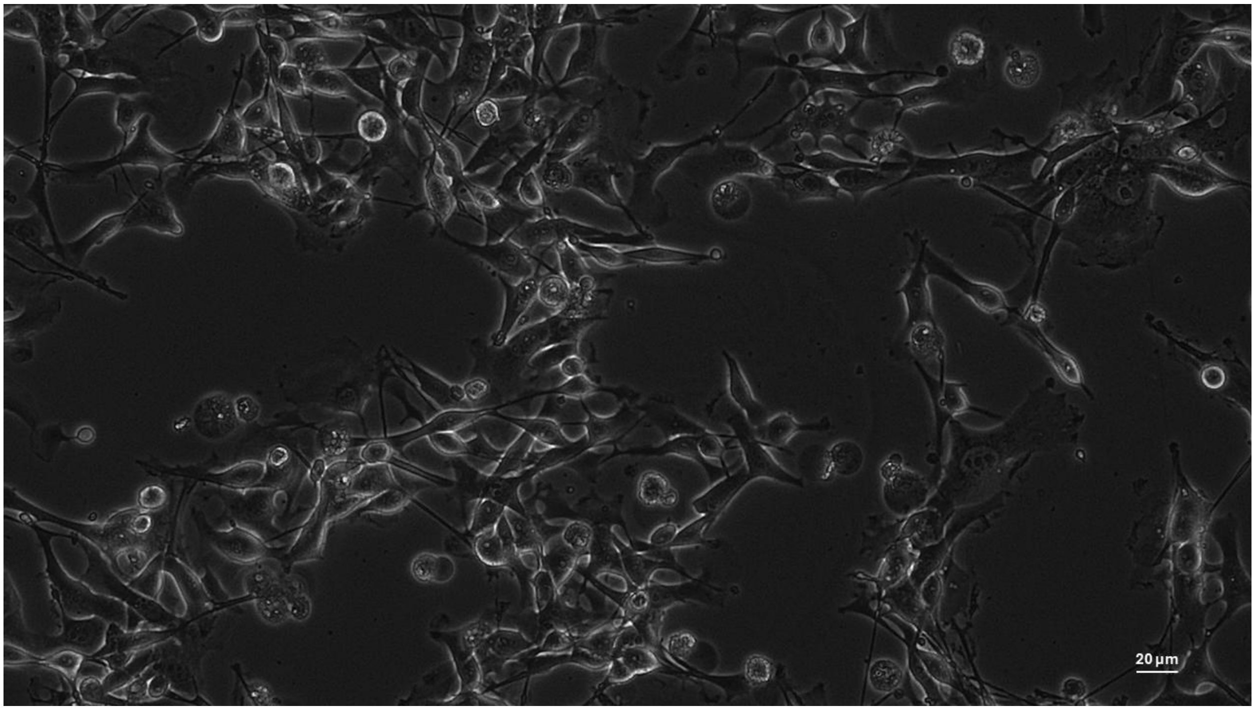

Figure S5. Morphology of B16-F10 murine melanoma cells treated with 10 µg malvidin-3-O-glucoside equiv-alents/mL extract of Fetească Neagră cv., scale bar = 20 µm.
